# Δ133p53α enhances metabolic and cellular fitness of TCR-engineered T cells and promotes superior antitumor immunity

**DOI:** 10.1136/jitc-2020-001846

**Published:** 2021-06-10

**Authors:** Kevin Jan Legscha, Edite Antunes Ferreira, Antonios Chamoun, Alexander Lang, Mohamed Hemaid Sayed Awwad, Gigi Nu Hoang Quy Ton, Danuta Galetzka, Borhane Guezguez, Michael Hundemer, Jean-Christophe Bourdon, Markus Munder, Matthias Theobald, Hakim Echchannaoui

**Affiliations:** 1 Department of Hematology, Oncology and Pneumology, University Medical Centre of the Johannes Gutenberg University Mainz, Mainz, Germany; 2 Department of Internal Medicine V, University of Heidelberg, Heidelberg, Germany; 3 Department of Radiation Oncology and Radiotherapy, University Medical Centre of the Johannes Gutenberg University Mainz, Mainz, Germany; 4 German Cancer Consortium (DKTK), Partner Site, Mainz, Germany; 5 School of Medicine, University of Dundee, Dundee, UK; 6 Research Center for Immunotherapy (FZI), University Medical Centre of the Johannes Gutenberg University Mainz, Mainz, Germany

**Keywords:** cell engineering, immunotherapy, adoptive, receptors, antigen, T-lymphocytes, costimulatory and inhibitory T-cell receptors

## Abstract

**Background:**

Tumor microenvironment-associated T cell senescence is a key limiting factor for durable effective cancer immunotherapy. A few studies have demonstrated the critical role of the tumor suppressor TP53-derived p53 isoforms in cellular senescence process of non-immune cells. However, their role in lymphocytes, in particular tumor-antigen (TA) specific T cells remain largely unexplored.

**Methods:**

Human T cells from peripheral blood were retrovirally engineered to coexpress a TA-specific T cell receptor and the Δ133p53α-isoform, and characterized for their cellular phenotype, metabolic profile and effector functions.

**Results:**

Phenotypic analysis of Δ133p53α-modified T cells revealed a marked reduction of the T-cell inhibitory molecules (ie, CD160 and TIGIT), a lower frequency of senescent-like CD57^+^ and CD160^+^ CD8^+^ T cell populations, and an increased number of less differentiated CD28^+^ T cells. Consistently, we demonstrated changes in the cellular metabolic program toward a quiescent T cell state. On a functional level, Δ133p53α-expressing T cells acquired a long-term proliferative capacity, showed superior cytokine secretion and enhanced tumor-specific killing in vitro and in mouse tumor model. Finally, we demonstrated the capacity of Δ133p53α to restore the antitumor response of senescent T cells isolated from multiple myeloma patients.

**Conclusion:**

This study uncovered a broad effect of Δ133p53α isoform in regulating T lymphocyte function. Enhancing fitness and effector functions of senescent T cells by modulation of p53 isoforms could be exploited for future translational research to improve cancer immunotherapy and immunosenescence-related diseases.

## Background

Adoptive cellular therapy using T cells engineered to recognize tumor-associated antigens or neoantigens following viral transfer and expression of T cell-receptor (TCR) encoding genes has advanced as a promising and personalized immunotherapy for chemorefractory leukemia and solid cancer.^1 2^ Encouraging results have been achieved in clinical trials using TCR transduced T cells. However, further improvements are needed to achieve durable responses.^3^ Potential obstacles of therapeutic efficacy are likely associated with ineffective trafficking and homing of T cells to and within the tumor.^2^ Moreover, multiple immunosuppressive mechanisms mediated by, for example, the interaction of programmed cell death ligand 1 (PD-L1) or poliovirus receptor (PVR, CD155) expressed on tumor cells with programmed cell death 1 (PD-1) and T cell immunoreceptor with Ig and ITIM domains (TIGIT) upregulated on T cells can counteract T cell effector functions.^4–6^ The development of antibodies blocking these negative regulators (immune checkpoints) represents a breakthrough in cancer immunotherapy and has led to durable responses in various cancer types.^7 8^ Ultimately, the tumor microenvironment (TME) drives T cells to an exhausted or senescent state with terminal differentiation characterized by poor proliferation and impaired antitumor responses.^9^ Engineering T cells that are less prone to tumor-driven dysfunction is therefore fundamental to improve antitumor responses in patients.

Cellular senescence displays a state of permanent proliferation arrest and is classified as either telomere-dependent (replicative senescence) due to a limitation of proliferative capacity^10^ or telomere-independent (premature senescence) due to external stimuli, such as oncogenic stress.^11^ Increasing numbers of senescent T cells are associated with many pathological conditions, such as infection and cancer.^12 13^ Moreover, senescent T cells accumulate during the normal lifespan of healthy individuals as well. These cells are mainly characterized by downregulation of the costimulatory molecule CD28 and increased expression of CD57,^13^ CD160, KLRG1, and exhibit an extenuated response to antigen stimulation.^14 15^ Although the mechanisms which regulate T cell senescence remain unclear, the tumor suppressor p53 emerged as a potential key player. *TP*53 has a crucial role in the maintenance of the genetic stability and, thus the prevention of cancer formation. It induces a number of cellular responses including DNA repair, cell cycle arrest and apoptosis. *TP*53 regulates the expression of many genes and is involved in cellular senescence.^16 17^ The finding that the human p53 gene contains an alternative promotor and transcribes multiple splice variants, resulting in the expression of 12 different protein isoforms p53 (α, β, γ), Δ40p53 (α, β, γ), Δ133p53 (α, β, γ), and Δ160p53 (α, β, γ)^18 19^ highlight the complexity of the p53 network. The human isoforms that are most associated with cellular senescence are the C-terminally and the N-terminally truncated p53β and Δ133p53α. The p53β protein isoform contains the transactivation domains (TAD) and the DNA-binding domain (DBD) but terminates with 10 additional amino acids, lacking half of the classic oligomerization domain, a nuclear export signal (NES) domain and the negative regulation domain. In contrast, Δ133p53α lacks both of the TAD and part of the second conserved region of the DBD but contains the NES. These two naturally occurring p53 isoforms act as physiological regulators of cellular proliferation and senescence in normal human fibroblasts, in human T lymphocytes,^13 20^ but also in human brain astrocytes and in induced pluripotent stem cells.^21 22^ In mice, the first report revealing that the p53 gene codes for more than one functional protein was published by Rotter and colleagues back in 1985,^23^ describing the presence of another p53 variant in transformed fibroblasts as a result of alternative splicing of intron 10, named p53AS (alternatively spliced). Later, MΔ41p53, the mouse counterpart of human Δ40p53α forms was isolated.^24^ In addition, a shorter N-terminal form produced by an internal promoter within the mouse p53 gene, MΔ157p53, equivalent to the human Δ160p53 form was identified. It was also shown that MΔ41p53 and MΔ157p53 can be expressed as a C-terminal AS variant.^25^ However, in contrast to human and high order primates, mice do not produce Δ133p53 isoforms or beta isoforms. Transgenic mice overexpressing Δ40p53 present an increased cellular senescence, a slower growth rate, and premature aging phenotype due to abnormal hyper-activation of the insulin-like growth factor signaling.^26^ Mechanistically, while human p53β can bind differentially to promoters and enhance expression of senescence-associated p53 target genes, Δ133p53α modulates gene expression and increases DNA-repair efficiency through interaction with full-length p53 and homologous p53 protein, p63 and p73.^27–29^ Δ133p53α also directly binds DNA on a novel type of p53-responsive element in enhancer and promoters to regulate gene transcription.^29^Δ133p53α also acts as a dominant negative inhibitor of senescence genes.^18 30^ Overexpression of Δ133p53α in near senescent human fibroblasts extends the cellular replicative lifespan by inhibiting the expression of p21^Waf1/Cip1^ and other p53 transcriptional target genes, including microRNA-34a. In contrast, overexpression of p53β induces cellular senescence by the upregulation of p53 target genes such as p21^Waf1/Cip1^ via cooperation with full-length p53.^13^ Although in T lymphocytes, the role of these two p53 isoforms as potential regulators of cellular senescence has been reported,^20^ their function in more complex and tissue-specific context, including immune-related disorders or cancer remains unexplored.

Here, we demonstrated for the first time that gene expression of the Δ133p53α isoform in tumor-antigen (TA) TCR-engineered T cells improves effector functions in vitro assays and in a mouse model of adoptive T cell transfer. Circumventing senescence in antigen receptor-redirected T cells by genetic modification with Δ133p53α provides a novel strategy to improve robustness and resilience of antitumor responses.

## Methods

### Animal studies

CyA2K^b^,^31^ OT-I and NOD.Cg-Prkdc^scid^IL2rg^tm1Wjl^/SzJ (NSG) mice were obtained from the central animal facility of the Johannes Gutenberg University Mainz, Germany. CyA2K^b^ and OT-I mice were used as source of donor splenocytes and NSG mice as recipient animals for tumor models. NSG mice were injected (s.c.) with osteosarcoma cell line Saos2/143 in the left flank and adoptively transferred (intravenous) with genetically modified human CD3^+^ T cells as previously published.^31^ Mice were sacrificed when the tumor size was 1 cm^3^ (or at termination of the study), and freshly isolated tumors and spleens were dissociated by mincing the tissue with scalpels into 0.5 mm small pieces. Dissociated tissue was further triturated and filtered through a 100 µm cell strainer to obtain single-cell suspension and analyzed by flow cytometry. Serum was obtained from the peripheral blood at the time of sacrifice.

### Blood samples, primary cells and cell lines

Buffy Coats from healthy donors were obtained from the Transfusion Center of the University Medical Center Mainz and the Institute for Immunology/IKTZ, Heidelberg University, Germany. Primary human T cells were obtained from the peripheral blood of patients with newly diagnosed multiple myeloma (MM). Peripheral blood mononuclear cells were isolated from peripheral blood via ficoll density gradient centrifugation. Tumor cell lines used in this study included the human HLA-A2.1^+^ p53 null osteosarcoma Saos2, its p53-transfectant Saos2/143^31^ and the C57BL/6-derived colon carcinoma MC38-OVA and melanoma B16-OVA. The A2K^b^ p53mutant MEF tumor cell line was described earlier.^31^ Human cell lines were maintained in RPMI 1640 supplemented with 10% heat inactivated fetal calf serum (FCS), 2 mM L-glutamine, 100 U/mL penicillin and 0.1 mg/mL streptomycin. Murine tumor cell lines were maintained in DMEM (GIBCO) supplemented with 10% FCS, 25 mM HEPES-buffer, 1% glutamine, and 1% penicillin-streptomycin.

### Peptides, antibodies and reagents

HLA-A2.1 restricted p53_aa264-272_ (LLGRNSFEV) was synthesized by Biosyntan (Berlin, Germany) and dissolved in DMSO. Antibodies used for Western blotting were mouse monoclonal antibodies (mAb) antihuman p53 (DO-2) sc-53394 (Santa Cruz Biotechnology, Heidelberg, Germany), antihuman p53 (HR231) MA1-12648 (Invitrogen), sheep antihuman p53 isoforms KJC12 (gift from Bourdon, Dundee, UK), rabbit mAbs anti-tri-methyl-Histone H3 (Lys4) (C42D8), anti-tri-methyl-Histone H3 (Lys9) (D4W1U) (Cell Signaling Technology), anti-HIF1a (Ab92498) (Abcam), rabbit mAb anti-Akt (pan) (C67E7) (Cell Signaling Technology, Cambridge), rabbit mAb anti-p21 (ab109520) (Abcam) and the mouse mAb anti-MDM2 (SMP14) sc-965 (Santa Cruz). Bafilomycin A1 (InvivoGen) was used as autophagy blocker.

### Genetic Modification of T Lymphocytes

cDNA encoding for human Δ133p53α and p53β (gift from Bourdon, Dundee, UK) were cloned into the retroviral vector pMx_Ires_puromycin (RTV-014, Cell Biolabs) via BamHI/NotI. As a TA model, we used our high-affinity sc p53TCR specific for the broadly expressed (non-mutant) HLA.A2.1-restricted p53_aa264-272_ peptide. The original scTCR scaffold was described earlier,^26^ and the cDNA-encoding sequence was further codon-modified (Invitrogen GeneArt, Regensburg, Germany) and cloned into one single bicistronic 2A-based retroviral vector.^24^ We used the pGMP retroviral vector encoding the scTCR^31^ for gene transfer in murine T cells. To redirect human T cells, the scTCR coding sequence was cloned into the retroviral vector pBullet_Ires_neomycin^32^ via NcoI/BamHI. Retroviral transduction, selection and expansion of human and mouse bulk CD8^+^/CD4^+^ T cells (with CD3/CD28-beads or by peptide-specific stimulation (K562_A2^+^_CD80^+^ pulsed with 10 µg p53 peptide) were performed as described earlier.^31 33^ Separation of human CD8^+^ T cell fractions (TIGIT^high^ and TIGIT^low^) was performed with MACS cell separation beads (Miltenyi Biotec) according to the manufacturer’s protocol.

### Flow cytometry analysis

Antibodies used in this study are listed in online supplemental table S1. Cells were stained according to the manufacturer’s instructions. To detect the expression of the lysosomal-associated membrane protein 1 (LAMP1/CD107a) (as a surrogate marker for degranulation) on the surface of CD8^+^ T-cells, effector cells were incubated for 24 hours with target tumor cells in the presence of monensin (Monensin Solution 1000X, eBioscience) and the CD107a antibody. Phorbol 12-myristate 13-acetate (PMA) + ionomycin (Sigma-Aldrich) were used as control inducers of CD107a expression. The determination of apoptotic cells was performed with a standard Annexin V and propidium iodide (PI) (BD Biosciences) staining according to the manufacturer’s instructions. TCR Vβ repertoire of TCR-engineered T cells in vivo was analyzed by flow cytometry with antibodies from the Beta Mark TCR Vβ Repertoire Kit (Beckman Coulter) directed against 19 individual TCR/Vβ chains. Flow cytometry acquisitions were performed on a FACSCanto II and BD FACSLyric (BD Biosciences), and data were analyzed with FlowJo_V10 software (Tree Star).

10.1136/jitc-2020-001846.supp1 Supplementary data

### T cell functional assays

T cell proliferation in vitro was assessed by carboxyfluorescein succinimidyl ester (CFSE) dilution assay. T cells were labeled with 5 µM CFSE for 5 min and subsequently stimulated with antigen-presenting cells as described before. Cells were analyzed at the indicated time points for CFSE dilution by flow cytometry. Standard 5h ^51^Cr-release assays were performed at the indicated effector-to-target (E:T) ratio in duplicate wells as described earlier.^31^ Long-term killing capacity of TCR-engineered T cells was determined by colony forming assay (CFA). Briefly, effector T cells were cocultured with antigen^+^ (Saos2/143) or antigen^-^ (Saos2) target tumor cells in 6- or 12-well plates at 37°C with 5% CO_2_ at the indicated E:T ratio. After 24 hours, T cells as well as non-adherent lysed tumor cells were washed out and if necessary, transferred to a second round of fresh tumor cells. The remaining adherent viable tumor cells were fixed with 4% PFA for 10 min at room temperature (RT) and subsequently stained with 1% crystal violet dye (Merck KGaA, Germany) for 15 min at RT. Crystal violet was washed off by adding Phosphate buffered saline (PBS) and the plate scanned for visual evaluation of colony counts. For quantitative analysis, the dye was dissolved by adding 5% SDS and the corresponding optical density (absorbance) measured at 570 nm using a microplate reader (Dynex MRX, Magellan BioScience). Replicative senescence of T cells in vitro was evaluated by calculating the population doubling levels (PDLs) using the following formula as described in^20^ : log_10_(number of cells after expansion) - log_10_(number of cells seeded)/log_10_2. Secreted cytokines/chemokines in vitro culture and in serum were determined by Luminex Multiplex Assays (MAGPIX) using Human Cytokine & Chemokine (34 plex) kit (eBioscience, San Diego, USA) according to the manufacturer protocol. For in vitro assays, effector T cells and target cells were cocultured in 12-well plates before for 24–48 hours as described for CFA and cytokine concentrations measured in the culture supernatant. Serum was diluted 1:5 before measurement.

### T cell metabolic assays

The oxygen consumption rate (OCR) and the extracellular acidification rate (ECAR) were measured using the Seahorse XFp Analyzer (Agilent). Culture Miniplates were coated, using Poly-D-Lysine (Sigma P6407). To each well, 50 µL of Poly-D-Lysine was added. After an incubation time of 1 hour at RT, the wells were rinsed with sterile water. XFp Sensor Cartridge wells were hydrated by adding 200 µl XF calibrant to each well and 400 µL to each outside well. Cartridges were then stored in a non-CO_2_ incubator at 37°C overnight. XFp Cell Energy Phenotype Test Kit (Agilent) was performed according to the manufacturers’ protocol. In detail, 1.0×10^6^ T cells per well were seeded in XF Base Medium (DMEM). The medium was supplemented with 10 mM glucose, 1 mM sodium pyruvate and 2 mM L-Glutamine and the pH was adjusted to 7.4. Using the Cell Energy Phenotype Test Kit, OCR and ECAR were measured under basal steady-state conditions (unstimulated T cells) and under stressed conditions. The stressed condition was induced by injection of 1 µM (final concentration) oligomycin and 1 µM (final concentration) fluoro-carbonyl cyanide phenylhydrazone (FCCP). The results were analyzed using the Seahorse Wave Desktop Software.

### Western blotting

Proteins were extracted from T cells using lysis buffer (Pepstatin, PMSF 100 mM, Sodium fluoride 1M, Sodium orthovanadate, Leup, 1% Brij 96 V solution) (Sigma-Aldrich). Protein concentration was determined by a DC Protein Assay (BioRad) according to the manufacturer’s recommendations. Western blotting was performed according to Bourdon’s (Dundee, UK) guidelines using reagents and devices from ThermoFisher Scientific. In brief, samples were loaded on a precast polyacrylamide gel (Bolt 10% Bis Tris Plus) and separated by electrophoresis. Samples were transferred to a nitrocellulose membrane (iBlot 2 Transfer Stacks) using the iBlot 2 Dry Blotting System. Membranes were blotted with the primary antibodies (listed above) overnight at 4°C and detected with anti-rabbit HRP-conjugated secondary antibody 7074S (Cell Signaling Technology, Cambridge, UK) (dilution 1:5000), anti-mouse HRP-conjugated secondary antibody sc-2005 (Santa Cruz Biotechnology, Heidelberg, Germany) (dilution 1:2000) anti-sheep HRP-conjugated secondary antibody ab7111 (Abcam, Cambridge, UK) (dilution 1:5000) for 1 hour at RT. Finally, chemiluminescence was detected with iBright Western Blot Imaging System after 5 min incubation with SuperSignal West Femto substrate (ThermoFisher Scientific) and 1 min exposure time.

### Quantitative real-time PCR

Total RNA was extracted from T cells with the RNeasy Mini Kit (Qiagen) according to the manufacturer’s protocol. RNA concentration was determined using the NanoDrop One (ThermoFischer Scientific). To generate cDNA, reverse transcription was performed with 2 µg of RNA and the High-Capacity cDNA Reverse Transcription Kit (ThermoFischer Scientific). Primers are listed in online supplemental table S2. The GeneTouch Thermal Cycler (Biozym Scientific) was used for reverse transcription with the following thermal conditions: 10 min at 25°C, 60 min at 37°C and 5 min at 85°C. Relative expression of transcripts was determined by quantitative real-time-PCR using the PowerUp SYBR Green master mix (Applied Biosystems) and QuantStudio 3 system (Applied Biosystems). The ΔΔC_t_ method was used according to the manufacturer’s protocol (Real-time PCR handbook, Applied Biosystems) to determine fold difference in expression normalized to the reference gene as indicated.

### Statistical analysis

Statistical analysis of differences between groups was determined by two-tailed Student’s t test or log-rank test for mouse studies using GraphPad Prism V.5.01.(^*^P<0.05, ^**^p<0.01,^***^p<0.005).

## Results

### Δ133p53α modulates the phenotype of CD8^+^ T cells toward a less senescent state

**Figure 1:**
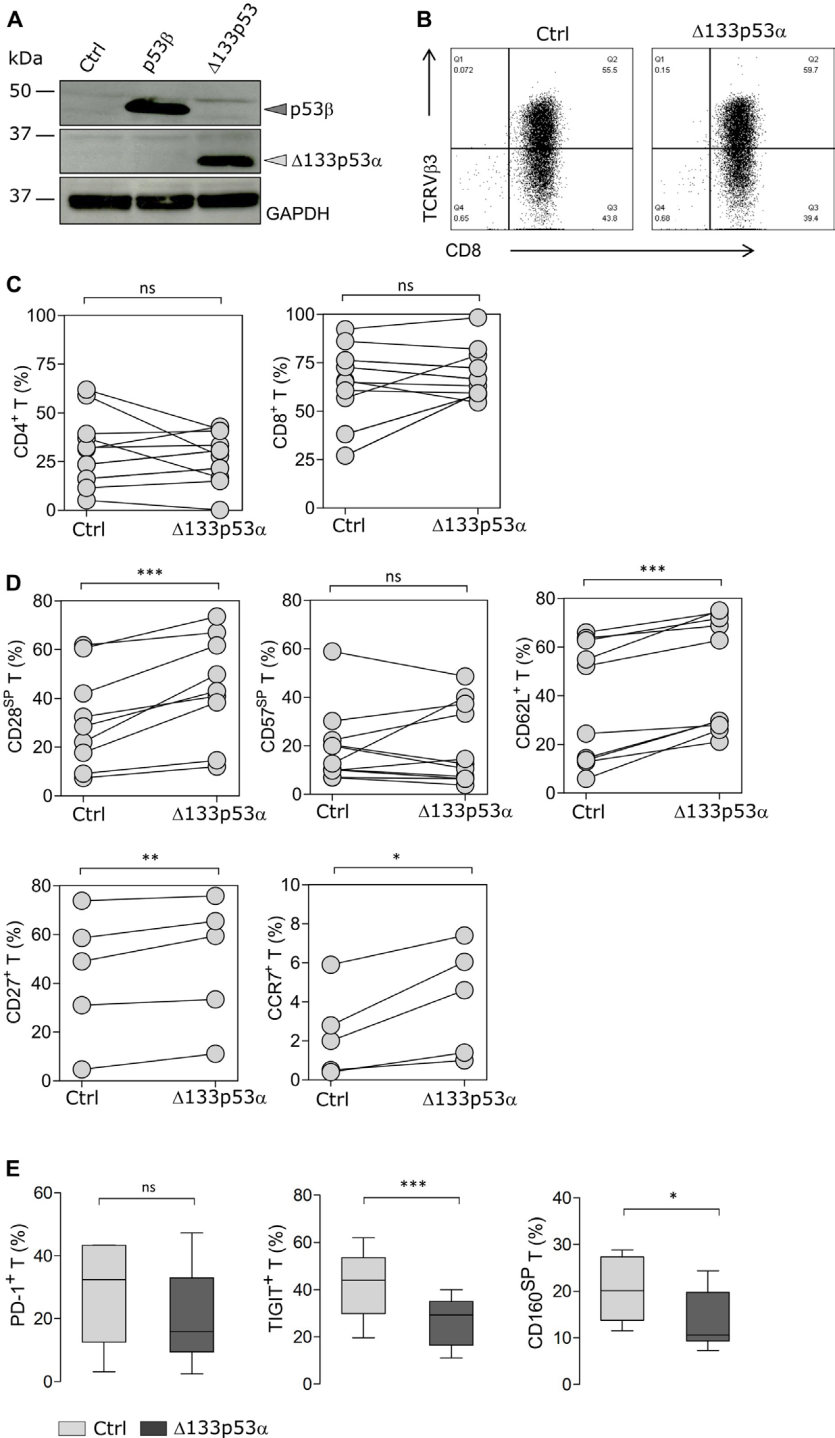
Δ133p53α modulates expression of exhaustion markers and inhibitory receptors on T cells. (A) Immunoblot confirming the overexpression of p53β and Δ133p53α isoforms after retroviral transduction and puromycin selection of human T cells. Empty vector was used for control cells. (B) Representative data for the cell surface expression of the transduced scTCR in CD8^+^ T cells, which was determined by flow cytometry using anti-TCRVβ3 mAb. (C) Percentage of CD8^+^ and CD4^+^ T cells for paired samples of Δ133p53α-modified or control T cells from healthy donors (n=12 biological replicates). (D) Flow cytometry data for cell surface expression of CD28, CD57, CD62L, CD27 and CCR7 for paired samples of Δ133p53α-modified or control T cells from different donors, shortly after transduction (n=5–16). Analysis of the cellular phenotype of T cells was carried out at early stage (1–3 weeks) after transduction. (E) Difference in PD-1 (p=0.1848), TIGIT and CD160 expression between control and Δ133p53α-transduced cells demonstrated in box plots. Error bars indicate SE of mean (SEM). SP stands for single positive. *P<0.05, **p<0.01, ***p<0.001, ns (not significant), by two-tailed Student’s t-test. scTCR, single-chain T cell-receptor.

T cells isolated from healthy donors, were retrovirally transduced with the p53 isoform Δ133p53α or empty vector (mock control) and characterized for proliferation, phenotype and major effector functions. Gene-modified T cells were selected by puromycin treatment. The overexpression of Δ133p53α was confirmed by western blot (figure 1A). The blot showing p53β expression was used as a control to validate the specificity of the antibodies directed against either the N-terminal or C-terminal domain of p53. Functional validation of the Δ133p53α isoform was confirmed by the downregulation of the p53 senescence-associated gene p21^Waf1/Cip1^ and the MDM2-master regulator of p53, as reported previously^13^ (online supplemental figure S1). Additionally, the cells were equipped with an antigen-specific TCR (scTCR),^31 33^ allowing us to study the effect of Δ133p53α on antigen-specific T cells. T cells coexpressing the p53 isoform and the scTCR were selected by puromycin + neomycin treatment. TCR expression as determined by cell surface staining with an anti-TCRVβ3 specific antibody was similar between Δ133p53α-overexpressing (57.6%) and control (51.5%) CD8^+^ T cells (figure 1B). Also, the frequency of CD8^+^ and CD4^+^ T cell subsets remained unchanged (figure 1C). CD8^+^ T cells were further characterized for the expression of differentiation markers by flow cytometry analysis. T cell populations with distinct phenotypes and functional properties can be identified by the expression/loss of CD28 and CD57 markers. During aging, the number of senescent-like CD28^-^CD57^+^CD8^+^ T cells increases, while the frequency of early-activated (non-differentiated) CD28^+^CD57^-^CD8^+^ T cells decreases. The frequencies of the CD28^+^CD57^-^ (referred to as CD28 single positive, SP) population and CD62L-expressing cells were significantly higher in Δ133p53α-T cells compared with control cells shortly after transduction. In addition, expression of the costimulatory molecule CD27 and the chemokine receptor CCR7, which are lost in differentiated T cells (ie, in effector memory or terminal effector phenotype) were increased in Δ133p53α-T cells. However, the percent of CD28^-^CD57^+^ (CD57SP) T cells remained comparable in both groups (figure 1D). Senescent or dysfunctional-T cells are also characterized by the expression of specific immune checkpoints. After several weeks of in vitro culture, the expression of inhibitory receptors was assessed. Among tumor-associated senescence factors and terminally differentiated markers, CD160 and TIGIT were expressed on a lower frequency in Δ133p53α-modified T cells (figure 1E), suggesting a less senescence phenotype. Of note, although the frequency of PD-1^+^CD8^+^ T cells was lower on expression of Δ133p53α, it did not reach significance (p=0.1848).

### Δ133p53α triggers metabolic reprogramming in T cells

**Figure 2:**
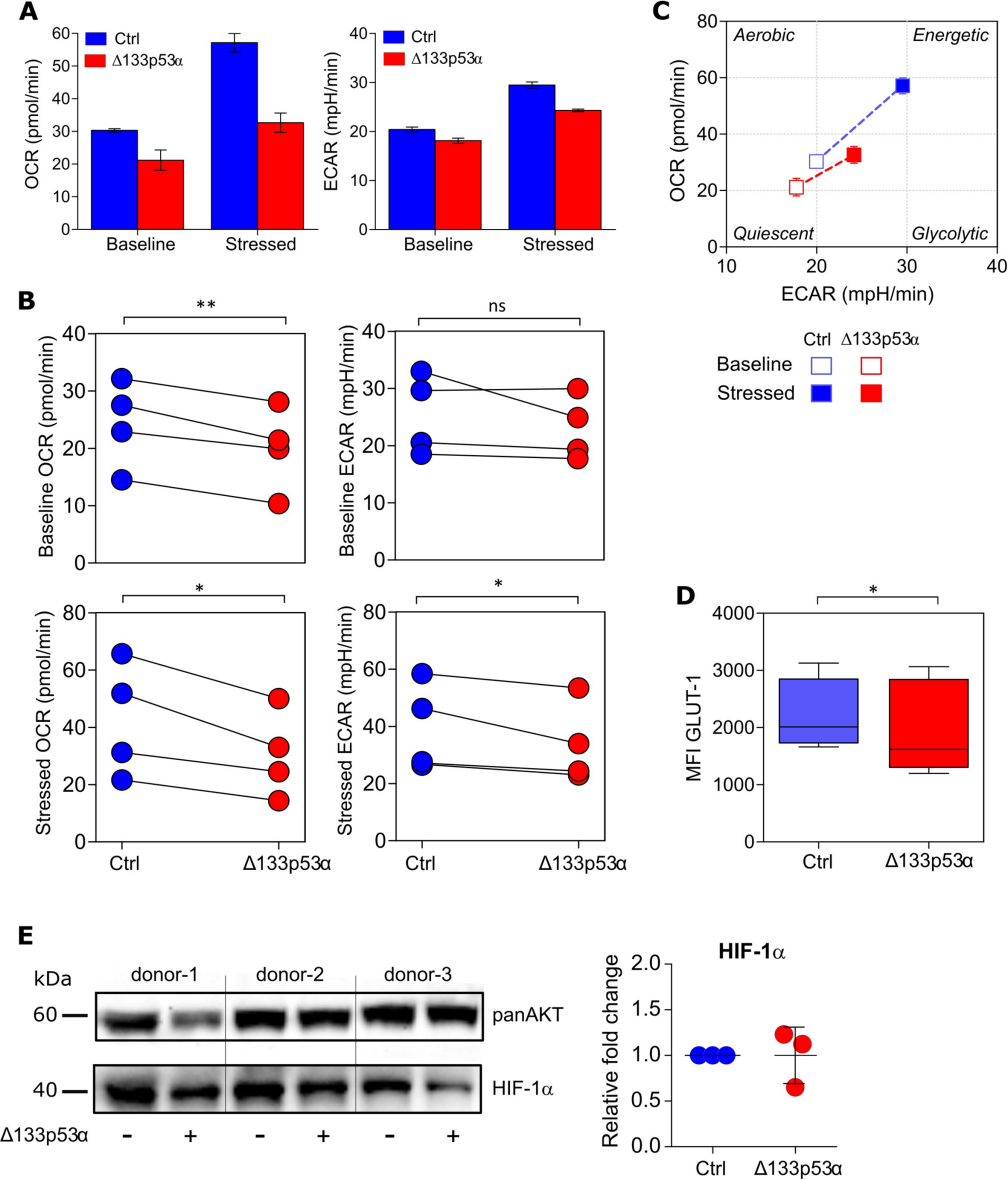
Δ133p53α dictates metabolic reprogramming in T cells. (A) Oxygen consumption rate (OCR, left figure) and extracellular acidification rate (ECAR, right figure) of Δ133p53α-transduced and control cells before (Baseline) and after (Stressed) addition of oligomycin and FCCP measured with a XFp Extracellular Flux Analyzer. Representative of n=4 biological replicates, shown as mean±SEM. (B). Dot plots depicting OCR and ECAR measures for paired samples of Δ133p53α-modified or control T cells from four healthy donors. (C) Energy Phenotype Profile of indicated cells demonstrating the relative utilization of glycolysis and mitochondrial respiration. (D) Box plots showing the expression level (as mean fluorescence intensity, MFI) of glucose transporter 1 (GLUT1) in control and Δ133p53α-transduced cells (n≥5). (E) Protein expression of HIF-1a in engineered CD8^+^ T cells from three healthy donors. Immunoblot (left) and dot plots (right) showing the fold change expression in Δ133p53α-modified as compared with control T cells. Analysis of the metabolic activity, including the expression of GLUT1 and HIF-1a were carried out early stage (1–3 weeks) after transduction. Error bars indicate SE of mean (SEM). *P<0.05, **p<0.01. ns, not significant.

Beside the cellular phenotype and differentiation status, T cell function relies on the uptake of nutrients and adequate energy production. Therefore, the metabolic program is adapted to meet the metabolic demands and functional needs.^34^ Resting cells, like naïve T cells, remain in a cellular and metabolic quiescent state.^34 35^ On antigen-recognition and activation, however, T cells increase aerobic glycolysis and mitochondrial oxidative phosphorylation (OXPHOS) activity for clonal expansion.^36 37^ To further characterize Δ133p53α-transduced T cells, we assessed their dynamic metabolic reprogramming by XFp Extracellular Flux analysis early after transduction. Under basal conditions, as well as after OXPHOS inhibition and mitochondrial uncoupling (=stressed conditions), Δ133p53α-T cells had a decreased glycolytic activity, measured by the ECAR and a reduced (OCR, indicator of OXPHOS) (figure 2A). Corresponding to the less differentiated cellular phenotype, the reduced activity of glycolysis and OXPHOS indicates a quiescent metabolic phenotype of Δ133p53α-T cells under basal conditions compared with control cells (figure 2B). These data provide evidence that Δ133p53α-overexpression does not only lead to changes in cellular phenotype, but is also associated with bioenergetic shifts toward a more quiescent metabolic phenotype (figure 2C), with the capacity to adapt their metabolic activity to the functional needs. To further, document these metabolic differences, we included the analysis of the glucose transporter 1 (GLUT1), which is also differently express in quiescent/naïve vs effector T cells.^38^ Concordantly, we observe a lower expression intensity in Δ133p53α-T cells as compared with controls (figure 2D), which correlate with their ‘quiescent-like’ phenotype. However, expression analysis of HIF-1a, as an additional factor in the metabolic transition to glycolysis, did not show a significant change (figure 2E). These results suggest the metabolic switch in Δ133p53α-T cells is most likely HIF-1a independent process.

### Overexpression of Δ133p53α invigorates T cell proliferation and improves effector functions of TCR-engineered T cells

**Figure 3:**
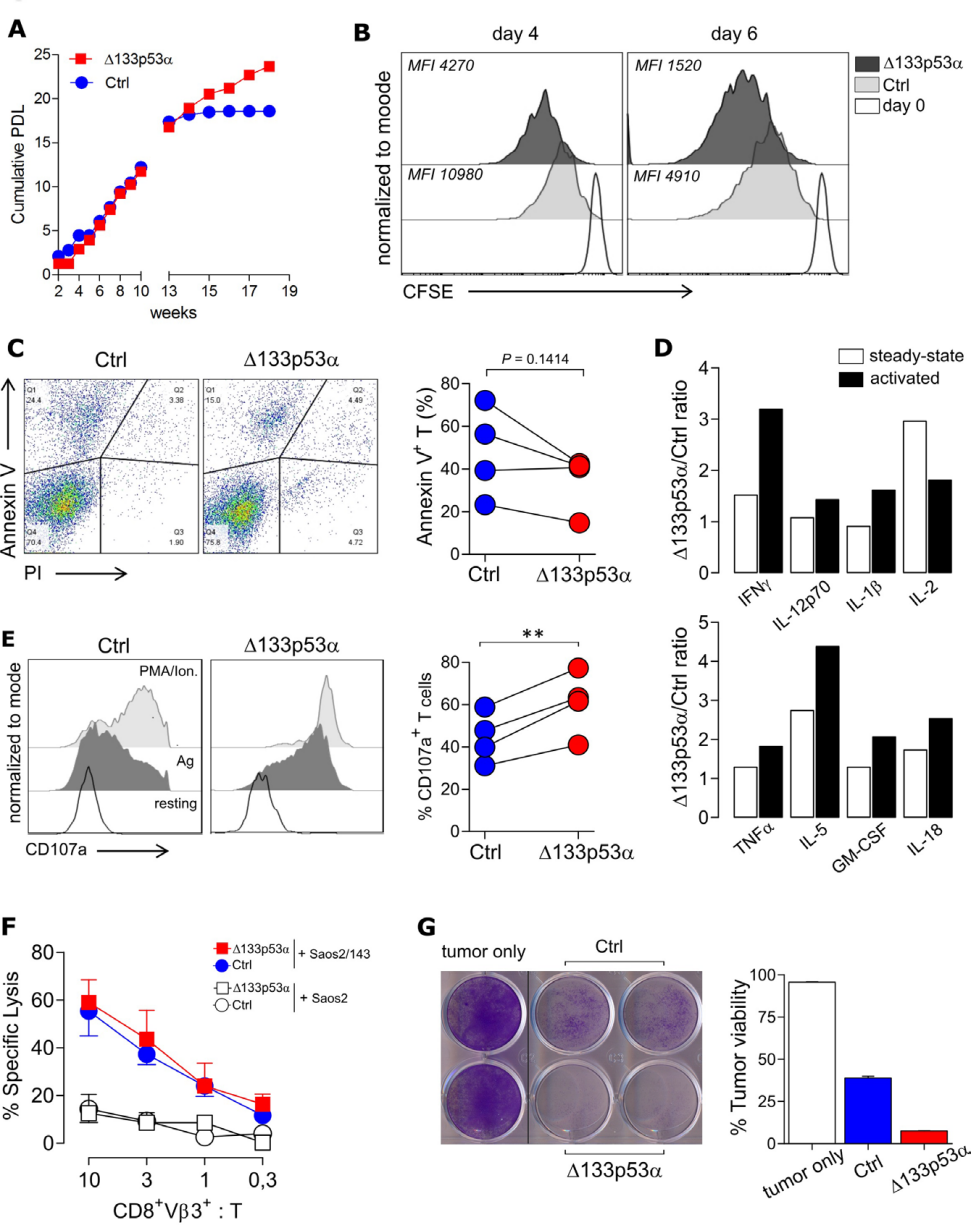
Δ133p53α invigorates T cell proliferation, cytokine response and improves long-term killing potential of antigen TCR-engineered T cells. (A) Cumulative PDL of control and Δ133p53α-transduced T cells over time from a representative donor. (B) CFSE proliferation assay of control and Δ133p53α-overexpressing T cells after several weeks of in vitro culture. Y-axis indicates the reduction of intracellular CFSE on Day 4 (left plot) and Day 6 (right plot) after antigen-specific stimulation (Day 0) and was normalized to mode. (C) Representative flow plots of the apoptosis marker Annexin V and Propidium Iodide (PI) for control and Δ133p53α-transduced T cells. Summary results are depicted as paired dot plots for each individual biological replicate (n=4). (D) Fold change of secreted cytokines from Δ133p53α-overexpressing to control cells under resting and activated conditions measured by Multiplex Immunoassay at late stage in vitro culture. For activation, T cells were cultured over 24 hours with target tumor cells Saos2/143 (E:T=1:1) (n=2 biological replicates). (E) Degranulation Assay of control and Δ133p53α-overexpressing T cells again under steady-state (=resting) and activated conditions (Ag=antigen-specific stimulation) at the same time point. Stimulation with PMA/Ionomycin was included as positive control. Degranulation is indicated by cell surface expression of LAMP1/CD107a, assessed by flow cytometry. Dot chart showing the percentage of CD107a^+^CD8^+^ T cells for paired samples of Δ133p53α-modified or control T cells from four healthy donors. (F) ^51^Cr-release Assay and (G) Tumor Colony-Forming Assay were used to evaluate short-term and long-term antitumor responses of Δ133p53α-modified compared with control T cells. For ^51^Cr-release assay-specific lysis of target (Saos2/143) and control (Saos2^p53null^) tumor cells is illustrated at the indicated effector to target ratio (CD8^+^Vβ3^+^:T). Tumor colony-forming assays were performed over 24 hours per round with an effector to target ratio (CD8^+^Vβ3^+^:T) of 1:1. Remaining tumor colonies were labeled with crystal violet dye. Quantification was performed by measuring the optical density (OD) of the residual dye, and values expressed as per cent of tumor viability. One representative experiment out of four biological replicates is shown. **P<0.01. CFSE, carboxyfluorescein succinimidyl ester; E:T, effector-to-target; IFNγ, interferon-γ; IL, interleukin; GM-CSF, Granulocyte-macrophage colony-stimulating factor; MFI, mean fluorescence intensity; TNFα, tumor necrosis factor-α.

In order to estimate the long-term proliferative capacity of Δ133p53α-T cells, we determined the PDL of aging cells in culture. Although cumulative PDL values were similar in the logarithmic phase, the proliferation index (or life-span) of control T cells reached a plateau around week 14, indicating a cellular senescence state, while Δ133p53α-T cells remained strongly proliferative (figure 3A). To confirm this finding, we compared the proliferation ability Δ133p53α-modified and control T cells at late timepoints of in vitro culture in a CFSE-based proliferation assay and noticed a higher cell division rate in Δ133p53α-T cells as demonstrated by lower mean fluorescence intensity (MFI) values of CFSE dye (figure 3B). In addition, the modified T cells exhibited a reduced apoptosis after stimulation, indicated by a lower (p=0.1414) frequency of Annexin V^+^PI^-^ cells (figure 3C). To further, evaluate effector functions of the near-senescent T cells, cytokine secretion, degranulation and cytotoxic capacity were examined. Cytokine profiles were determined under steady-state conditions, and after activation with TA. Secretion levels of cytokines, with potent effector or stimulatory effect were elevated in Δ133p53α-overexpressing T cells (up to fourfold) compared with control T cells, particularly after antigen activation (figure 3D). Raw values are depicted in online supplemental figure S2. We further evaluated the capacity of modified T cells to mobilize lytic granules, analyzing the expression of the (LAMP1/CD107a). The data show a strong degranulation response (indicated by an increased expression of CD107a) of Δ133p53α-modified T cells and control T cells after TA encounter (=Ag) as compared with ‘residual’ response under steady-state (=resting) condition (figure 3E, left plot). Importantly, the percent of CD107a^+^ T cells is significantly higher in Δ133p53α-modified T cells (figure 3E, right plot). To validate these observations, we assessed the cytolytic activity of Δ133p53α-engineered T cells against target tumor cells. Interestingly, the killing capacity was similar in both groups in a short-term (4–6 hours) lytic assay (figure 3F), yet, on repetitive coculture with tumor target cells over 24 hours, Δ133p53α-T cells showed a remarkable stronger elimination of tumor cells compared with control cells (25% vs 40% of viable tumor colonies, figure 3G). In sharp contrast to Δ133p53α, p53β expression in T cells was associated with induction of premature senescence, as documented by shorter lifespan, lower CD28 and higher CD57 expression, higher apoptosis levels and low cytolytic activity (online supplemental figure S3). However, as opposed to Δ133p53α data, the expression of the immune inhibitory molecule TIGIT (and to a lesser extent PD-1) showed a trend toward higher levels.

### TIGIT expression is antigen-dependent and affects TCR-mediated T cell cytolytic activity

**Figure 4:**
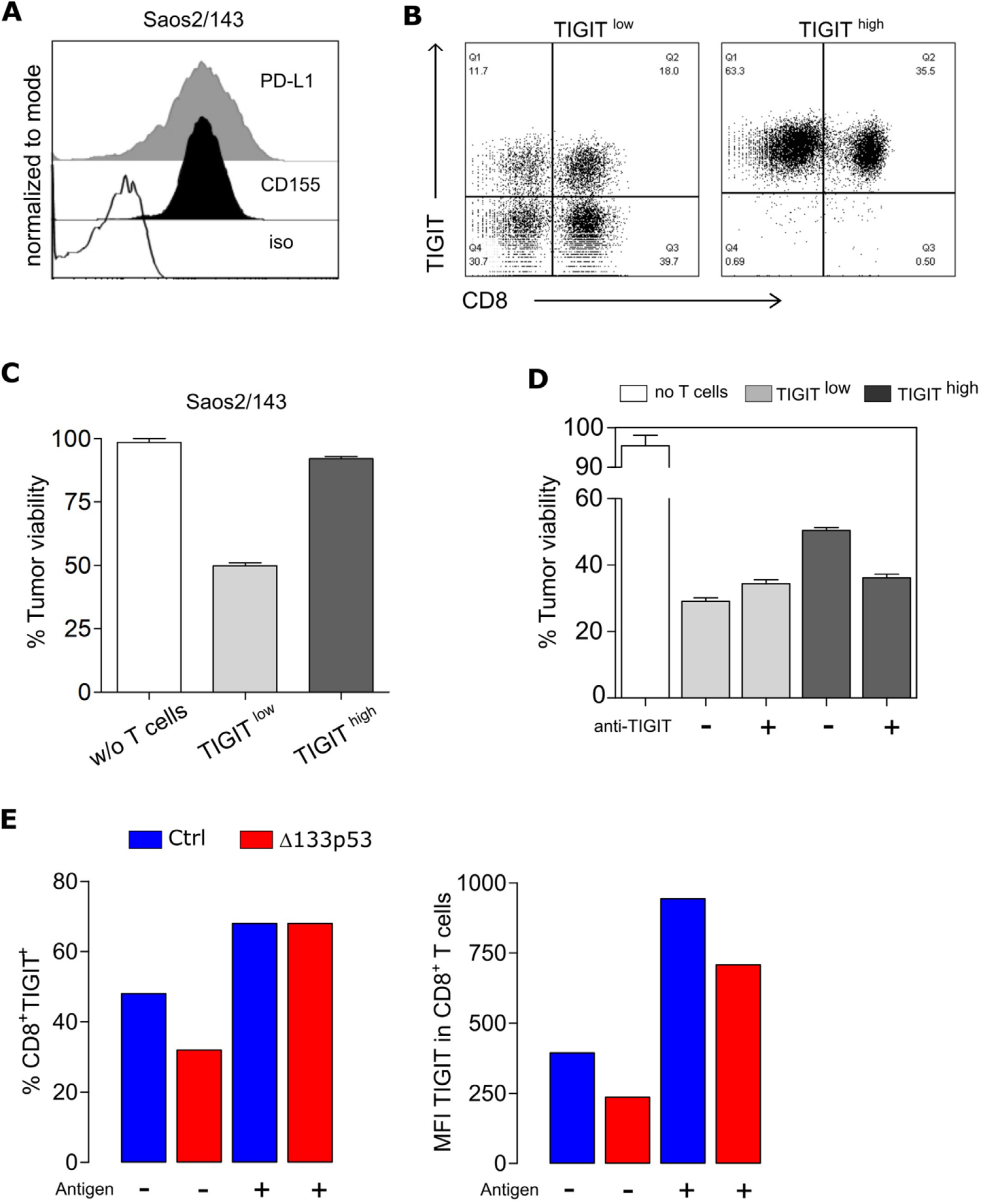
TIGIT induction is antigen-dependent and affects TCR-mediated T cell cytolytic activity. (A) Expression levels of PD-L1 and CD155 in the target tumor cell line Saos2/143 determined by flow cytometry. (B) Flow Cytometry Data showing TIGIT expression of CD8^+^ T cells after MACS-separation into TIGIT^low^ and TIGIT^high^ population. (C) Quantified killing capacity of TIGIT^low^ and TIGIT^high^ T cells was determined in a tumor colony-forming assay, and is expressed as the percentage of remaining viable tumor cells after coculture with effector T cells. (D) Quantified killing capacity of TIGIT^low^ and TIGIT^high^ T cells on TIGIT blockade as determined in a tumor colony-forming assay. (E) Upregulation of TIGIT on CD8^+^ T cells before and after antigen recognition via coculture with Saos2/143. Percentage (left) and MFI (right) was assessed by flow cytometry. Results from one representative experiment out of three biological replicates are shown. MFI, mean fluorescence intensity; PD-L1, programmed cell death ligand 1.

TIGIT is a central marker of T cell dysfunction and has been shown to be upregulated on human tumor-infiltrating CD8^+^ T cells (also regulatory T cells and NK cells) in a variety of cancers. Furthermore, TIGIT signaling can also alter T cell metabolism, as reported in patients with cancer.^38^ We then addressed the relevance of the reduced TIGIT expression in Δ133p53α-modified T cells in a functional in vitro assay. As a target tumor model, we used the osteosarcoma cell line Saos2/143 that naturally express the ligands for TIGIT (CD155/PVR) but also other inhibitory receptors, such as PD-L1 (figure 4A). scTCR-transduced T cells were separated into two fractions, CD3^+^TIGIT^high^ and CD3^+^TIGIT^low^ populations (figure 4B), and compared for their cytolytic activities. As expected, TIGIT^high^ T cells showed a reduced cytolytic capacity as compared with TIGIT^low^ T cells (figure 4C), which could be restored on TIGIT blockade (figure 4D, online supplemental figure S4A). TIGIT/CD155 engagement can inhibit T cell function directly by affecting TCR signaling^39^; however, the mechanism by which TIGIT upregulation is triggered remained unclear. Here, we show that after encountering target tumor cells, the percent of TIGIT-expressing T cells increases to similar levels in control and Δ133p53α-transduced cells, however, the expression level of TIGIT per cell (as determined by MFI values) remained reduced in Δ133p53α-T cells as compared with control cells (700 vs 950) (figure 4E). TIGIT upregulation is cell-cell contact mediated and antigen-dependent process, as coculture with Ag^+^ Saos2/143 target, but not Ag^−^ Saos2 cells, triggers an increase in TIGIT expression (online supplemental figure S4B). This mechanism was confirmed using tumor cell-free supernatant experiments (online supplemental figure S4B) and p53-pulsed Saos2 cells (online supplemental figure S4C). In an attempt to understand the mechanism by which Δ133p53α overexpression correlates with lower TIGIT expression, we examined this pathway at the transcriptional level. Comparative analysis of TIGIT mRNA revealed lower transcripts in Δ133p53α-T cells in comparison with control cells (online supplemental figure S5A), suggesting a transcriptional regulation. To validate these data in a ‘gene-unmodified’ experimental model, we stabilized Δ133p53α expression in control T cells by preventing its degradation by autophagy using the ATPase inhibitor bafilomycin A1^20^. We demonstrated that the level of TIGIT transcripts as well as TIGIT expression was severely reduced in T cells after treatment with bafilomycin A1 (online supplemental figure S5A, B). Additionally, we assessed the wide changes in histone modifications that might results from the overexpression of Δ133p53α isoform and affecting transcription at promoter sites for both H3K4me3 (active marks) and H3K9me3 (repressive marks). By testing three different biological T-cell donors, we observed a slight increase in H3K4me3 without noticeable change in H3K9me3 (online supplemental figure S5C), suggesting a minor focal increase in promoter activation and gene expression in Δ133p53α-overexpressing T cells.

### Expression of Δ133p53α in murine T cells is associated with reduced TIGIT levels and improved T-cell effector function

**Figure 5:**
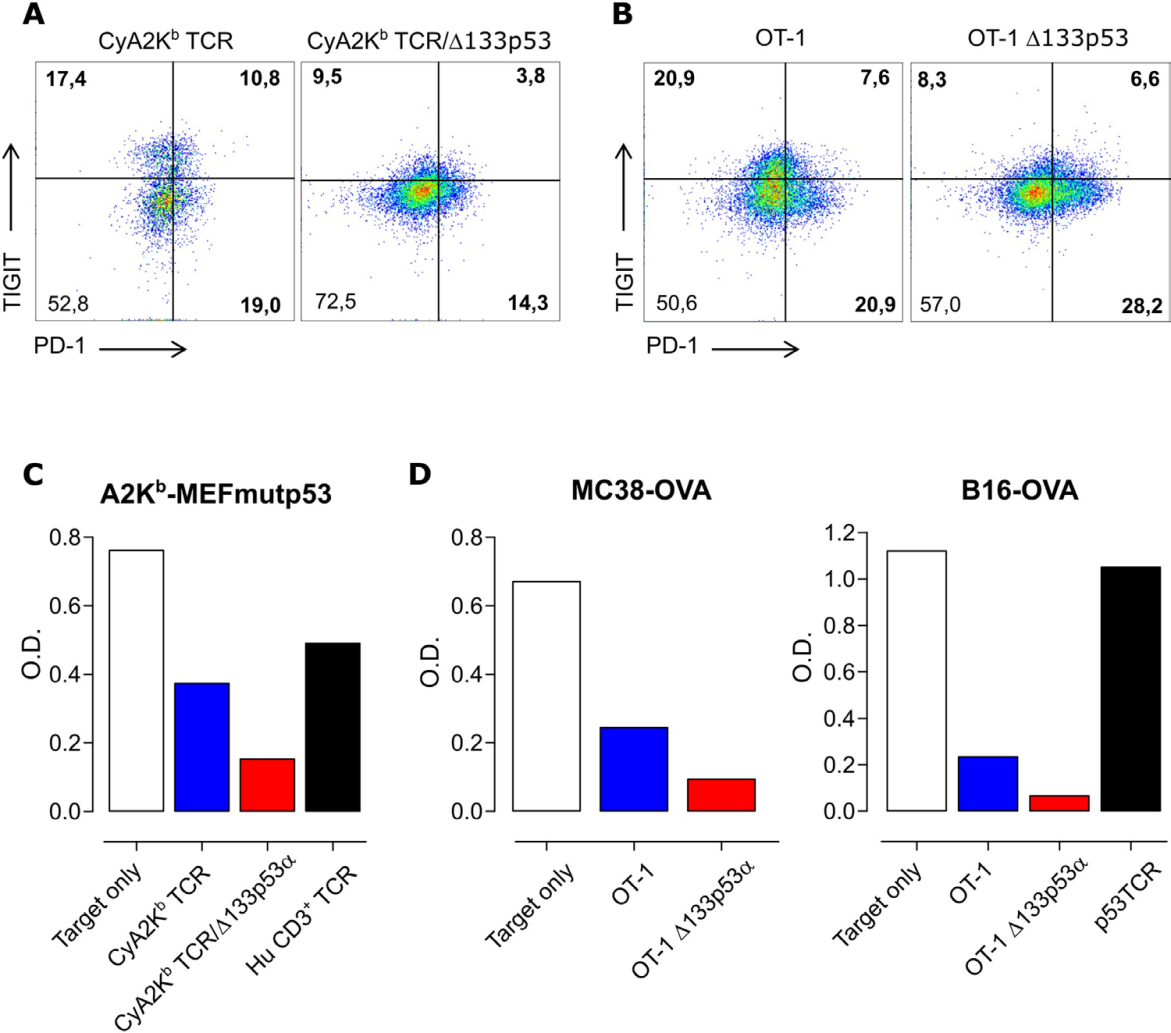
Expression of Δ133p53α in murine T cells is associated with reduced TIGIT levels and improved T-cell effector function. (A) Representative, flow cytometric data of TIGIT and PD-1 expression of mouse T cells obtained from CyA2K^b^ mice, transduced with scTCR and of T cells obtained from OT-I mice (B). Both were transduced with human Δ133p53α or empty vector. Quantitative data from a representative tumor colony-forming assay using CyA2K^b^ TCR mouse T cells and A2K^b^MEF^mutp53^ (C). For OT-I cells, MC38^OVA^ and B16^OVA^ served as target cells (D). The optical density (O.D.) indicates the amount of remaining viable tumor colonies after incubation with effector T cells. PD-1, programmed cell death 1; TCR, T cell-receptor.

To further confirm and validate our findings, we tested whether the major effects of Δ133p53α demonstrated in human CD8^+^ T cells could be observed in murine T cells. Therefore, we cotransduced T lymphocytes from CyA2K^b^ mice with a scTCR^31^ and the human Δ133p53α isoform or a mock control. As in human T cells, Δ133p53α-transduced CyA2K^b^/TCR^+^ mouse T cells exhibited reduced levels of TIGIT compared with control cells (figure 5A). Similar results were obtained in Δ133p53α-modified T cells from OT-I mice (figure 5B). Similar to human T cells, Δ133p53α did not affect PD-1 expression levels in murine T cells (figure 5A, B). In addition, these models demonstrated that Δ133p53α-modified CyA2K^b^ (figure 5C) or OT-I (figure 5D) murine T cells were able to eradicate target cells expressing high levels of CD155/PVR (online supplemental figure S6) more efficiently than control cells, consistent with the results of Δ133p53α in human T cells.

### Adoptive transfer of TCR/Δ133p53α-equipped T cells results in superior antitumor response in vivo

**Figure 6:**
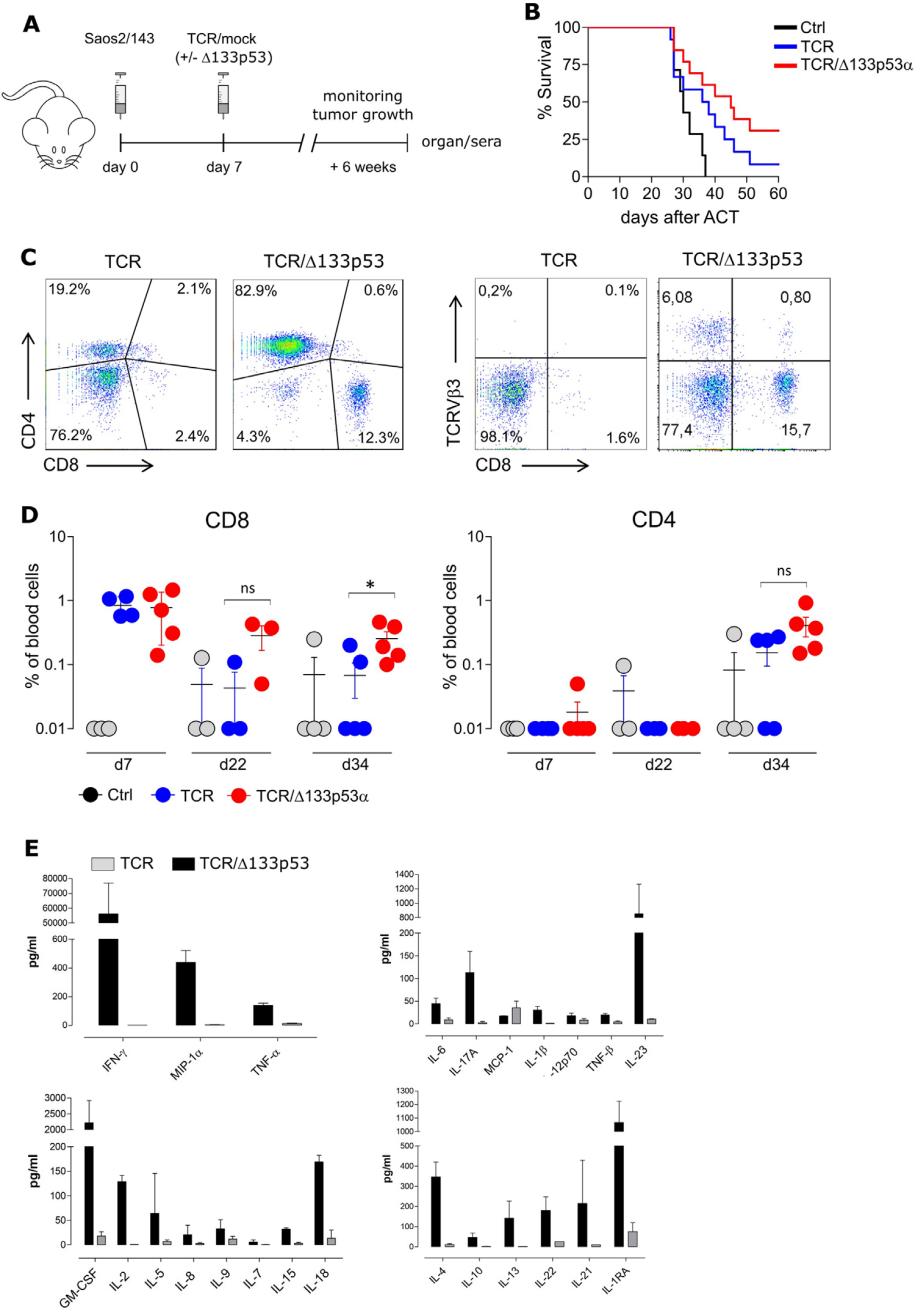
Adoptive transfer of TCR/Δ133p53α-equipped T cells results in superior antitumor response but is associated with cytokine release syndrome. (A) Schematic representation of the experimental model. NOD-scid IL2rg^null^ (NSG) were injected subcutaneously with Saos2/143 on day 0. T cells and IL-2 were injected intravenous on day 7 as described in the Methods. (B) Survival curves for mice treated with mock-control- (Ctrl), TCR-modified or TCR/Δ133p53α-modified T cells (pooled data of three individual experiments), n=32 mice. Significance between the animal groups was determined by log-rank test (p=0.0199). (C) Frequency of CD8^+^ and CD4^+^ T cells in the peripheral blood at days 7, 22 and 34 after adoptive transfer. *P<0.05, ns (not significant). (D) Representative data from Multiplex-Immunoassays using serum from NSG mice injected with TCR^+^ or TCR^+^/Δ133p53α^+^ T cells. Serum was collected after 6 weeks, before the mice were sacrificed. ACT, adoptive cellular therapy; IFNγ, interferon-γ; IL-6, interleukin 6; GM-CSF, Granulocyte-macrophage colony-stimulating factor; TCR, T cell-receptor; TNF, tumor necrosis factor.

Next, we assessed the antitumor efficacy of Δ133p53α antigen-specific TCR-modified T cells in a xenograft tumor model. Immunodeficient NSG mice were injected with Saos2/143 tumor cells, and infused with genetically modified-T cells (figure 6A). Δ133p53α-transduced T cells exhibited an improved antitumor response as reflected by a significantly prolonged survival of tumor bearing mice (figure 6B). A no transfer (PBS) experiment, to exclude any unspecific effect of mock-control T cells on tumor growth showed similar survival outcome as the control group (online supplemental figure S7A). Analysis of the starting T cell population showed comparable phenotype (CD4/CD8 ratio, TCRVβ3 and CD45RA/CCR7 expression levels) in Δ133p53α- and control-T cells at the time of infusion (online supplemental figure S7B–D). Of note, superior tumor control in Δ133p53α-treated mice was occasionally accompanied with signs of graft-versus-host disease (GvHD), which was characterized by loss of fur, reduced activity and weight loss. The development of GvHD was unlikely due to TCR mispairing-associated toxicity as we have used in this study an optimized scTCR with proven safety profile in vivo.^31^ This prompted us to further correlate the onset of GvHD in these animals with the kinetics of the Δ133p53α-T cell response. Analyses of spleens revealed a massive infiltration of human CD4^+^ and CD8^+^ T cells in TCR/Δ133p53α mice as opposed to TCR control group (figure 6C, online supplemental figure S7E). Concordantly, Δ133p53α-T cells demonstrated a longer engraftment in vivo as documented by a higher frequency of CD8^+^ and CD4^+^ T cells in the peripheral blood at days 34 after adoptive transfer (figure 6D). The frequency of antigen-specific TCR(Vβ3)-infiltrating T cells was also higher, yet not significant in the spleen and tumor-tissue of TCR/Δ133p53α animals (online supplemental figure S7E). As anticipated, while the starting T cell population infused in mice was mainly composed of naïve and effector T cells, persistent T cells in vivo exhibited rather an effector memory phenotype. Comparative analysis of sera collected from both animal groups shortly before sacrifice, revealed markedly increased levels of pro-inflammatory and immunomodulatory cytokines and chemokines in mice treated with Δ133p53α-T cells (figure 6E). These data are concordant with a previous study showing that Δ133p53α triggers proinflammatory cytokine response, including interleukin 6 (IL-6), IL-1β and IL-8 (via inhibition of p53 and induction of NF-κB) in a *Helicobacter pylori* infection model or cancer model.^40 41^

Further analysis of the TCR repertoire in spleen-infiltrating Δ133p53α-T cells revealed a polyclonal TCRβ signature (online supplemental figure S7G), indicating a diversity of responsive T cells in vivo.

### Reverting T cell senescence of MM patients by Δ133p53α gene transfer

**Figure 7:**
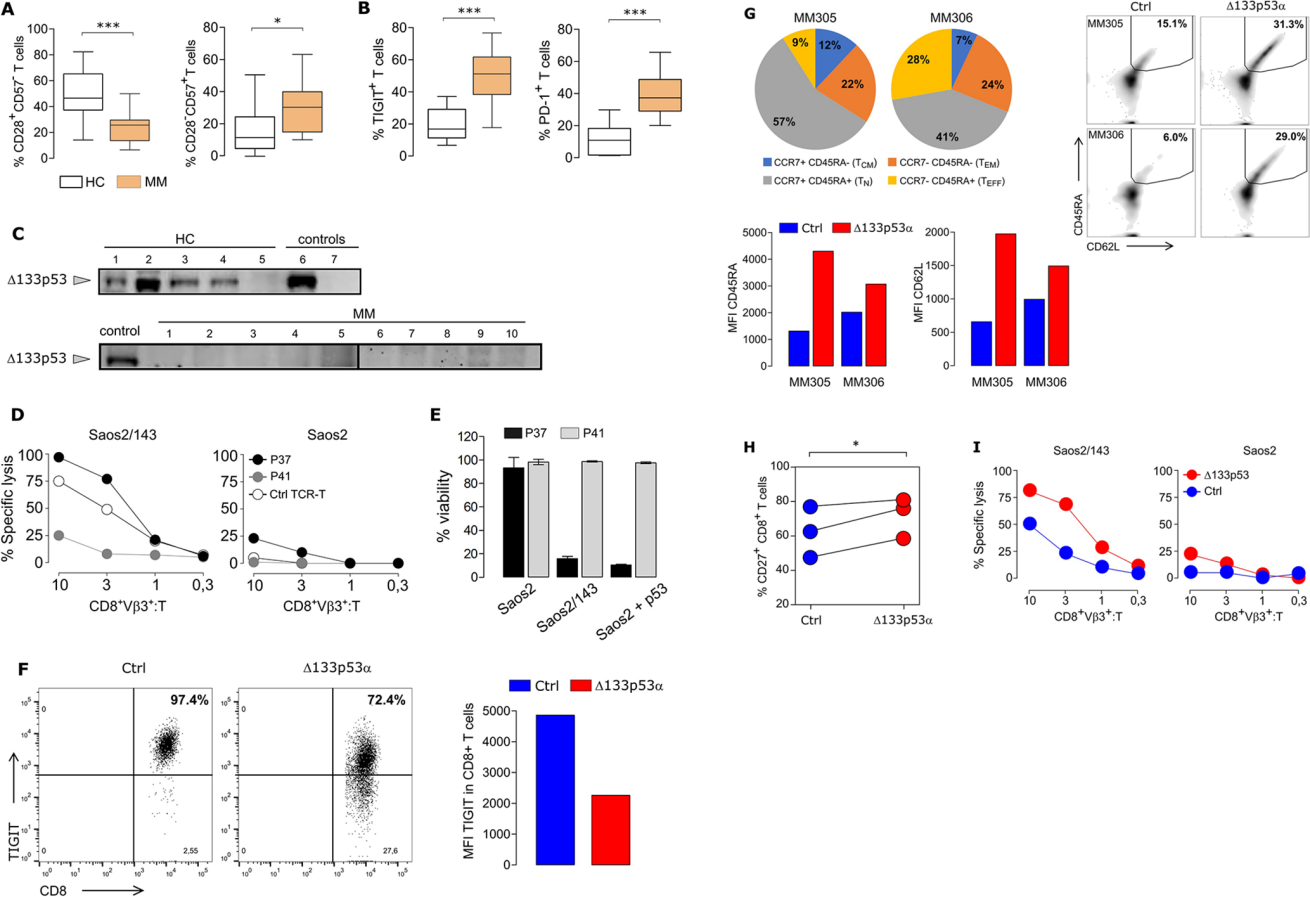
Reverting senescence in multiple myeloma (MM) patient T cells by Δ133p53α gene transfer. (A) Reduced frequency of CD8^+^CD28^+^CD57^-^ (left, p=0.0014) and increased frequency of CD8^+^CD28^-^CD57^+^ (right, p=0.0461) T cells from MM patients (n=10) compared with healthy donor T cells (HC, n=15). (B) T cells from MM patients (n=27) revealed high levels of TIGIT and PD-1 expression compared with healthy donor T cells (HC, n=7–15) (p<0.0001). (C) Western Blot showing protein expression of Δ133p53α in T cells from MM patients and healthy controls (HC). Δ133p53α-transduced T cells and Saos2^p53null^ (lane 6–7) served as positive and negative controls. Total protein loaded per lane was 32–60 µg for HC and MM samples. (D) Short-term and (E) Long-term Killing Assays comparing T cells from two MM patients with low TIGIT expression (P37, CD8^+^TIGIT^+^: 21%) and high TIGIT expression (P41, CD8^+^TIGIT^+^: 77%). (F) Overexpression of Δ133p53α in T cells from a MM patient (MM305) resulted in reduced frequency of TIGIT-expressing T cells (flow plota) and lower expression levels (MFI plot). (G) Phenotype of CD8^+^T cells from two myeloma patients (MM305, MM306) prior (pie chart, left) and after (flow plots, right) retroviral transduction with ctrl or Δ133p53α. Expression intensity of CD45RA and CD62L expression in CD8^+^ engineered T cells are shown as MFI plots. (H) Dot chart showing the percentage of CD27^+^CD8^+^ T cells for paired samples of Δ133p53α-modified or control T cells from three MM patients. (I) Short-term Killing Assays of T cells from the same patient (MM305) transduced with Δ133p53α or control vector exhibited an improved cytolytic response by Δ133p53α-overexpression at different effector (CD8^+^Vβ3^+^) to target (T) ratios. Error bars indicate SE of mean (SEM). *P<0.05, ***p<0.001, by two-tailed Student’s t-test. MFI, mean fluorescence intensity; PD-1, programmed cell death 1; TCR, T cell-receptor.

To further examine the in vivo relevance of Δ133p53α in the regulation of T cell senescence, peripheral blood T cells from newly diagnosed MM patients were phenotyped. MM patients had a reduced number of ‘naïve’ CD8^+^CD28^+^CD57^-^ and an increased frequency of senescent-like CD8^+^CD28^-^CD57^+^ single positive (SP) T cell subsets (figure 7A), suggesting a senescent state of CD8^+^ T cells in MM patients. Interestingly, analysis of the fraction of total CD28^+^ and CD57^+^ in CD8^+^ T cells showed similar expression profiles (online supplemental figure S8). Furthermore, we observed a markedly increased expression of the T cell-associated senescent markers TIGIT and PD-1 in MM patients (figure 7B). We then anticipated a reduced expression of the Δ133p53α isoform in MM as compared with healthy individuals. Protein expression data confirmed high levels of Δ133p53α-isoform in healthy individuals, while marginal or no expression was detected in MM patient T cells (figure 7C). Next, we evaluated the senescence status of MM CD8^+^ T cells in functional assays. T cells from two patients (P37 and P41) with distinct TIGIT, CD28SP and CD57SP expression profiles were selected. P41 and P37, characterized as TIGIT^high^CD57SP^high^CD28SP^low^ and TIGIT^low^CD57SP^low^CD28SP^mild^, were genetically equipped with a scTCR and tested for their antigen specific response. In both, short-term (figure 7D) and long-term (figure 7E) killing assays, P41-, but not P37-modified T cells, exhibited a severely impaired cytotoxic capacity against target Saos2/143 tumor cells. To explore the possibility to reprogram senescent T cells of MM patients into more ‘juvenile’ and effective T cells, we performed Δ133p53α gene transfer experiments in MM T cells. In line with our data from healthy individuals, Δ133p53α overexpression was associated with more than a twofold reduction in TIGIT expression (figure 7F). While the starting CD8^+^T cell population from two myeloma patients (MM305, MM306) prior transduction was mainly composed of naïve, effector memory (CD45RA-CCR7-) and effector (CD45RA+CCR7-) phenotype, the phenotype following transduction with TCR control vector showed a dominant effector memory and a low percentage (6% and 15%) of CD45RA+CD62L+ (naive). The frequency of naïve population as well as the expression intensity of both CD45RA and CD62L markers increased (twofold and fivefold) after overexpression of the Δ133p53α isoform (figure 7G). In line, Δ133p53α-modified MM T cells exhibit a higher expression of the costimulatory molecule CD27 (figure 7H). Importantly, Δ133p53α gene transfer in MM patient T cells promoted superior specific cytolytic activity against Saos2/143 tumor cells (figure 7I).

## Discussion

Senescence-induced T cell dysfunction in the TME impedes the clinical efficacy of cancer immunotherapy.^9 14^ An age-dependent accumulation of senescent T cells in healthy individuals which was associated with changes in expression of T cell surface markers and p53 isoforms has been described. In this study, p53β and Δ133p53α were identified as potential opposite markers for cellular senescence by providing evidence that overexpression of Δ133p53α could restore the proliferation capacity of late differentiated or senescent T cells in vitro.^20^ However, it remained unclear whether and how these isoforms can modulate TA-specific T cell functions, in particular in more complex in vivo tumor suppressive environment.

Here, we demonstrated that gene expression of Δ133p53α is associated with a metabolic switch and profound functional changes in TA-specific cytotoxic CD8^+^ T cells in vitro and in mouse tumor model. Phenotyping of T cells revealed a shift toward a less differentiated state characterized by an upregulation of CD28, CD27 and CD62L and a downregulation of key inhibitory receptors, CD160 and TIGIT. Accordingly, we observed a prolonged long-term proliferative potential and improved effector functions as demonstrated by enhanced tumor-specific cytolytic activity and a superior cytokine response in vitro. In contrast, p53β expression in T cells drives premature senescence phenotype, characterized by a lower CD28 and higher CD57 expression, higher apoptosis levels along with a shorter life-span.

T cells can modulate their cellular metabolism according to their functional needs.^34^ However, this metabolic switch can be altered by the TME which can lead to T cell dysfunction.^42^ Along with the induction of a less differentiated phenotype and enhanced effector functions, our results showed that Δ133p53α is associated with a metabolic reprogramming in tumor-reactive engineered CD8^+^ T cells characterized by a quiescent metabolic state with lower glycolytic activity and expression of GLUT1. This is in line with a recent study reporting that CD8^+^ T cells with a low mitochondrial membrane potential (ΔΨm) are enriched in CD62L^+^ central memory T cells, accompanied with a reduced glycolytic activity and mitochondrial respiration leading to enhanced proliferative capacity and increased antitumor activity in vivo.^43^ The reduced metabolic activity early after transduction may be adapted to low metabolic demands, which may be increased for rapid expansion after activation.

T cell metabolism can be altered in patients with cancer through TIGIT/CD155 signaling.^44^ Moreover, TIGIT/CD155 interaction is also involved in the suppression of T cell activation.^5 45^ TIGIT was found to be upregulated on human tumor-infiltrating lymphocytes^44 46^ and its blockade improved CD8^+^ T cell effector functions.^5 47^ Moreover, CD8^+^ T cells from aged healthy donors, exhibited a high TIGIT expression, accompanied by impaired effector functions.^48^ Here, we demonstrated for the first time the effect of Δ133p53α on TIGIT expression in antigen-specific T cells at the mRNA and protein levels. Additionally, T cells with high TIGIT expression had a compromised cytolytic response against CD155-expressing target cells. Consistently, T cell dysfunction in patients with MM^14^ has been recently attributed, among other factors, to high TIGIT expression.^47 49^ Accordingly, TIGIT blockade could restore anti-myeloma T-cell function.^47^ In the present study, we could confirm the senescent phenotype and high expression of TIGIT in T cells from newly diagnosed MM patients and further revealed a reduced expression of Δ133p53α. In line with our results from healthy donors T cells, TIGIT^high^-expressing MM T cells showed impaired antitumor responses which could be restored on Δ133p53α gene transfer. Improved T cell function of Δ133p53α-modified MM T cells was associated with a less differentiated phenotype characterized by de novo expressions of CD45RA, CD27 and CD62L. Thus, these results further demonstrate the capacity of Δ133p53α to reinvigorate effector functions of cancer patient-derived senescent T cells. On a more translational level, a lower expression of the senescence-associated and negative immune receptor TIGIT in antigen-TCR CD8^+^ T cells overexpressing Δ133p53α is novel (as it has not yet been reported) and has potential clinical relevance. TIGIT is emerging as the third (after CTLA-4 and PD-1) clinical target in immuno-oncology. Currently, more than 20 clinical trials testing anti-TIGIT agents (as single treatment or in combination with PD-(L)1 blockade) are launched by lead biopharmaceutical companies (such as Roche, BMS and Merck), including five phase 3 studies in solid cancers (glioblastoma, melanoma, lung, breast cancer, …) and hematological malignancies (MM).

Using a preclinical tumor model, we observed enhanced antitumor responses in Δ133p53α-T cells leading to superior overall survival of treated mice. This improved antitumor immunity was occasionally accompanied by a severe inflammatory response, confirmed by elevated levels of secreted cytokines in the serum and massive infiltration of T cells in the spleen. These findings suggest a cytokine-associated toxicity resulting from rare hyperactivated T cells induced by Δ133p53α, consistent with the profound proinflammatory phenotype reported in transgenic mice expressing a Δ133p53α-like isoform.^50^ These data calls for a safety approach to limit excessive immune response associated with sustained expression of Δ133p53α in engineered T cells. These potentially severe side effects caused by strong immune activation may be resolved with monoclonal antibodies against certain cytokines (like Tocilizumab), or by using a safety switch approach like an inducible caspase 9 suicide system.

In conclusion, our data demonstrated that Δ133p53α isoform acts as a potent enhancer of robustness and resilience in human cytotoxic T cells, which may represent a novel approach to improve T-cell-based cancer immunotherapies.

10.1136/jitc-2020-001846.supp2 Supplementary data

## Data Availability

All data relevant to the study are included in the article or uploaded as supplementary information.
