# Supplementary material for: Δ133p53α enhances metabolic and cellular fitness of TCR-engineered T cells and promotes superior antitumor immunity
Source: J Immunother Cancer. 2021 Jun 10;9(6):e001846. doi: 10.1136/jitc-2020-001846 (PMC8194333; doi:10.1136/jitc-2020-001846)
Supplement: Supplementary data [file jitc-2020-001846supp001.pdf]

jitc-2020-001846.R2

Supplemental Information

Δ133p53α enhances metabolic and cellular fitness of TCR-engineered T cells and promotes superior antitumor immunity

Authors: Kevin J. Legscha[1], Edite A. Ferreira[1], Antonios Chamoun[1], Alexander Lang[1], Mohamed HS. Awwad[2], Gigi NHQ Ton[2], Danuta Galetzka[3], Borhane Guezguez[1,4,5], Michael Hundemer[2], Jean-Christophe Bourdon[6], Markus Munder[1,7], Matthias Theobald[1,4,7], Hakim Echchannaoui[1,4]

Affiliations: [1]Third Department of Medicine, University Cancer Center (UCT), University Medical Center (UMC) of the Johannes Gutenberg University
[2]Department of Internal Medicine V, University of Heidelberg
[3]Department of Radiation Oncology and Radiotherapy, UMC of the Johannes Gutenberg University
[4]German Cancer Consortium (DKTK), Partner Site Frankfurt/Mainz, Mainz, Germany
[5]German Cancer Research Center (DKFZ), Heidelberg, Germany
[6]School of Medicine, University of Dundee, Dundee, UK
[7]Research Center for Immunotherapy, UMC of the Johannes Gutenberg University Mainz, Germany

Correspondence to: Hakim Echchannaoui | Tel: +49 6131 17-9722 | Email: echchann@uni-mainz.de

Legscha KJ, *et al. J Immunother Cancer* 2021; 9:e001846. doi: 10.1136/jitc-2020-001846

jitc-2020-001846.R2

Supplemental data

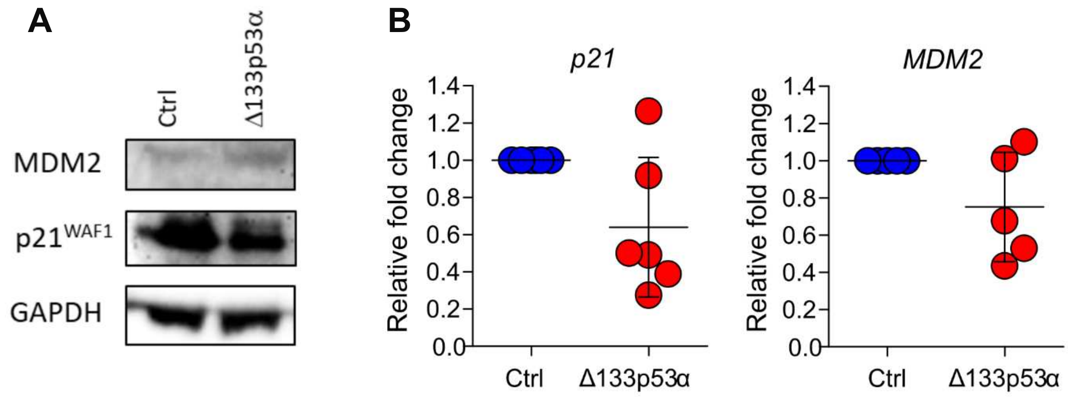

Figure S1. Δ133p53α modulates the function of the full-length p53. (A) Immunoblot analysis of the expression of p21$^{Waf1/Cip1}$ and MDM2 as p53 target genes in human CD8^{+} T cells transduced with an empty control (ctrl) vector or retroviral vector encoding for Δ133p53α isoform. (B) Summary data from 5-6 heathy are shown. Data were normalized to GAPDH and shown as relative fold change to Ctrl samples.

jitc-2020-001846.R2

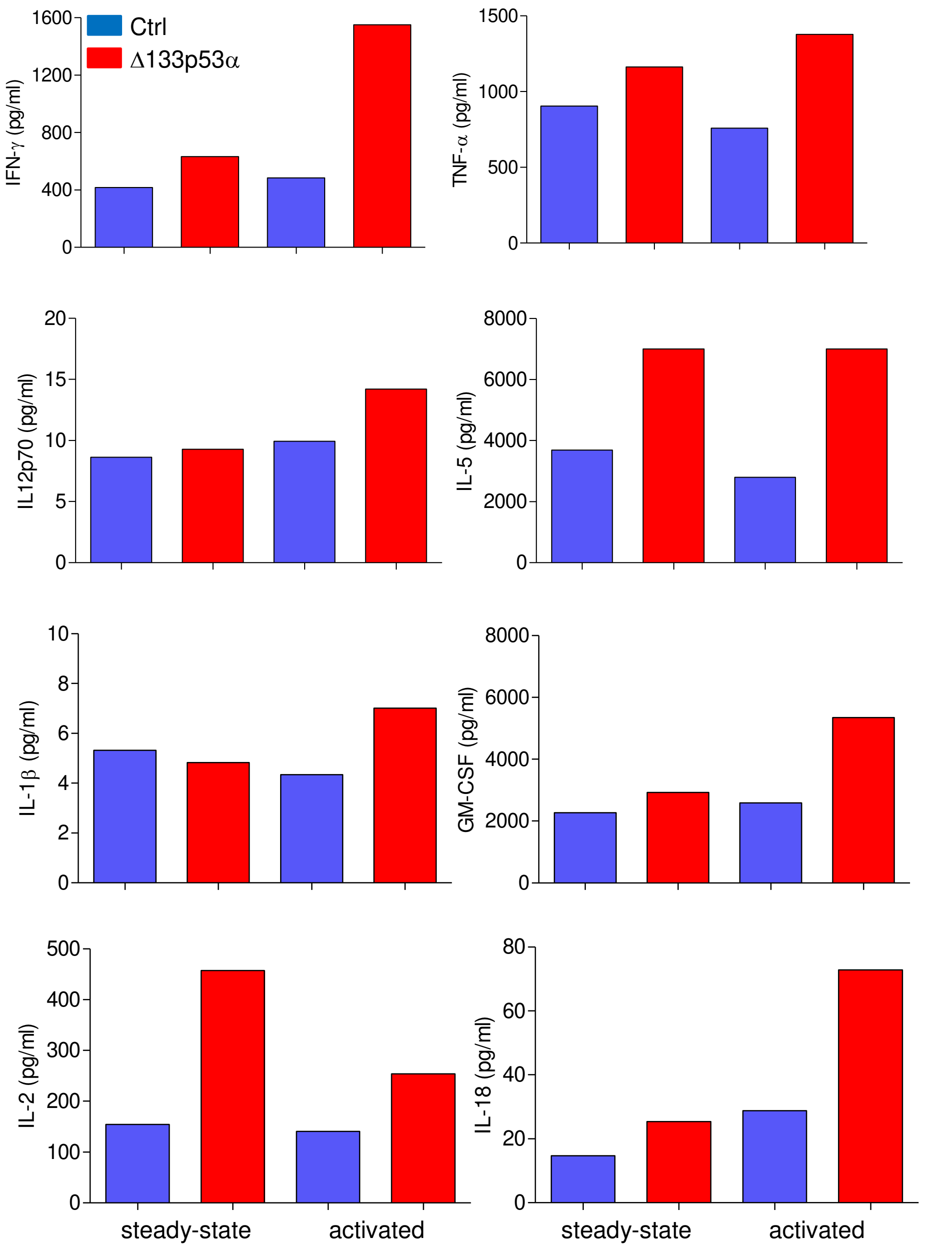

Figure S2. Δ133p53α invigorates cytokine response of antigen TCR-engineered T cells. Cytokine profiles of Δ133p53α-overexpressing and control T cells under steady-state (=resting) and after tumor antigen encounter (=activated) as measured by Multiplex Immunoassay at late stage in vitro culture. For activation, T cells were cultured over 24 hours with target tumor cells Saos2/143 (E:T = 1:1).

Legscha KJ, *et al. J Immunother Cancer* 2021; 9:e001846. doi: 10.1136/jitc-2020-001846

jitc-2020-001846.R2

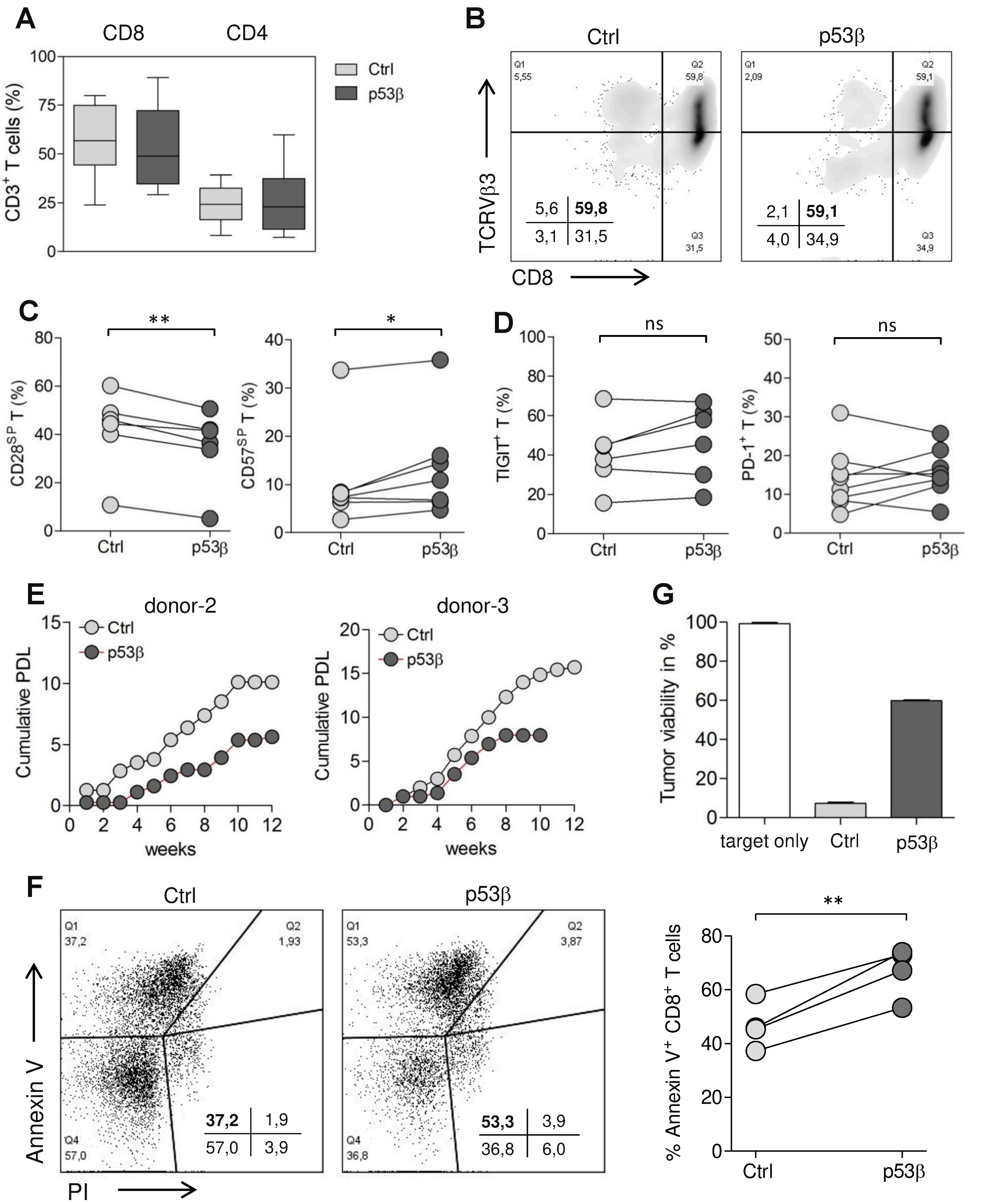

Figure S3. Characterization of p53β-driven senescence in engineered human CD8⁺ T cells. (A) Percentage of CD8⁺ and CD4⁺ T cells for paired samples of p53β-modified or control T cells from healthy donors (n=9 biological replicates). (B) Representative flow plots for the cell surface expression of the transduced scTCR in CD8⁺ T cells, which was determined by flow

cytometry using anti-TCRVβ3 mAb. (C) Flow cytometry plots depicting cell surface expression of CD28 and CD57 single positive (SP) for paired samples of p53β-modified or control $CD8^+$ T cells from different donors (n≥6). (D) Difference in TIGIT and PD-1 expression between control and p53β-transduced cells shown for each individual donor (n≥6). (E) Cumulative PDL of control and p53β-transduced $CD8^+$ T cells over time from two representative donors. (F) Representative flow plots of Annexin V and Propidium Iodide (PI) staining for control and p53β-transduced T cells, and the corresponding graphs depicting individual donors (n=4). (G) Long-term cytolytic activity of p53β-modified compared to control T cells as determined by Tumor Colony-Forming Assay (see Methods section). One representative experiment out of four biological replicates is shown. *P < 0.05, **P < 0.01, ns (not significant), by two-tailed Student's *t* test.

jitc-2020-001846.R2

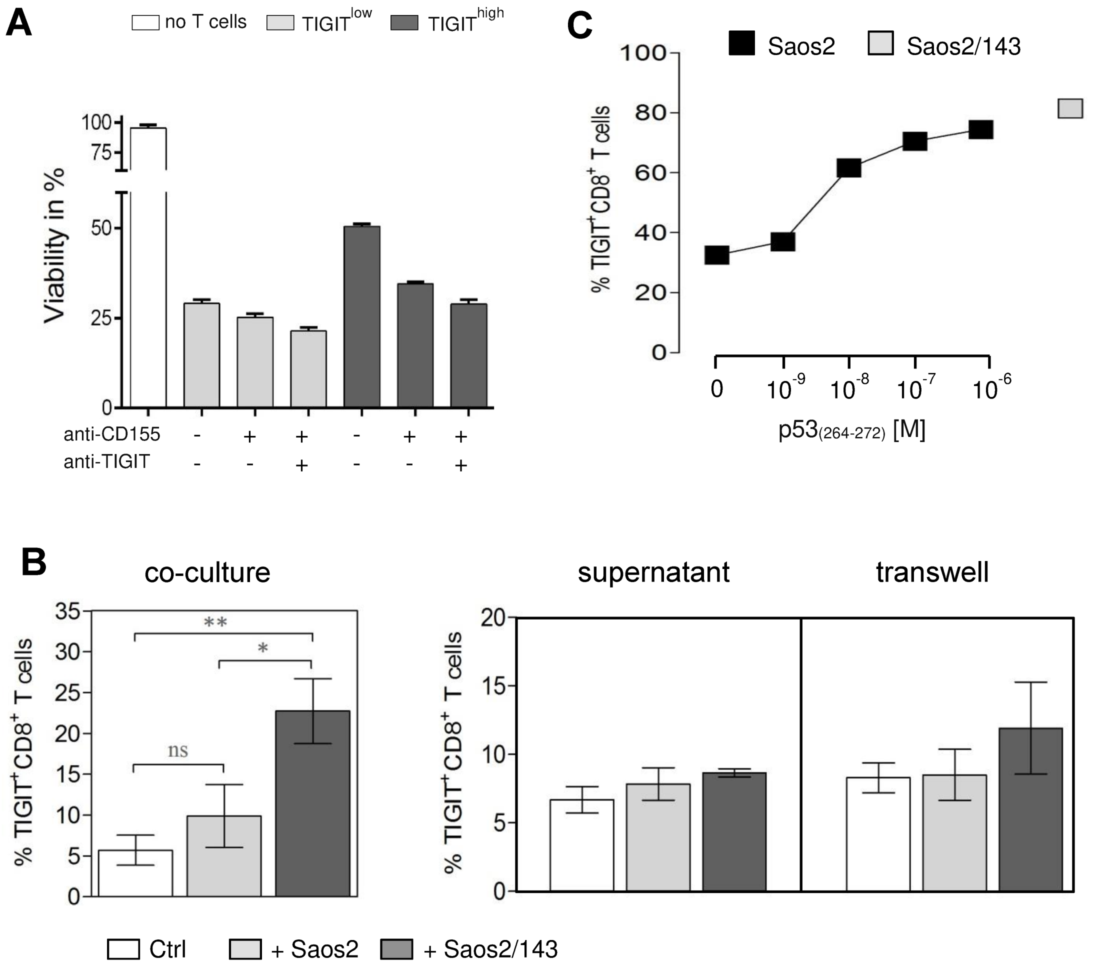

Figure S4. TIGIT expression in T cells is antigen-dependent and affects TCR-mediated cytolytic response. (A) Effect of CD155 and/or TIGIT blockade on the cytolytic activity of $TIGIT^{low}$ and $TIGIT^{high}$ TCR-effector T cells against Saos2/143 target tumors, as determined by tumor colony-forming based killing assays. (B) Flow cytometric data of TIGIT expression with or without incubation in cell-free supernatant collected from $Saos2^{p53null}$ or Saos2/143. TIGIT expression of T cells co-cultured with $Saos2^{p53null}$ or Saos2/143 under normal conditions or in transwell system. (C) TIGIT expression of $CD8^+$ T cells after co-culture with $Saos2^{p53null}$, which were pulsed with titrated concentrations of the target antigen ($p53_{264\text{-}272}$ peptide). n ≥ 3 biological replicates. Error bars indicate standard error of mean (SEM). *$P < 0.05$, **$P < 0.01$, ns (not significant), by two-tailed Student's *t* test.

jitc-2020-001846.R2

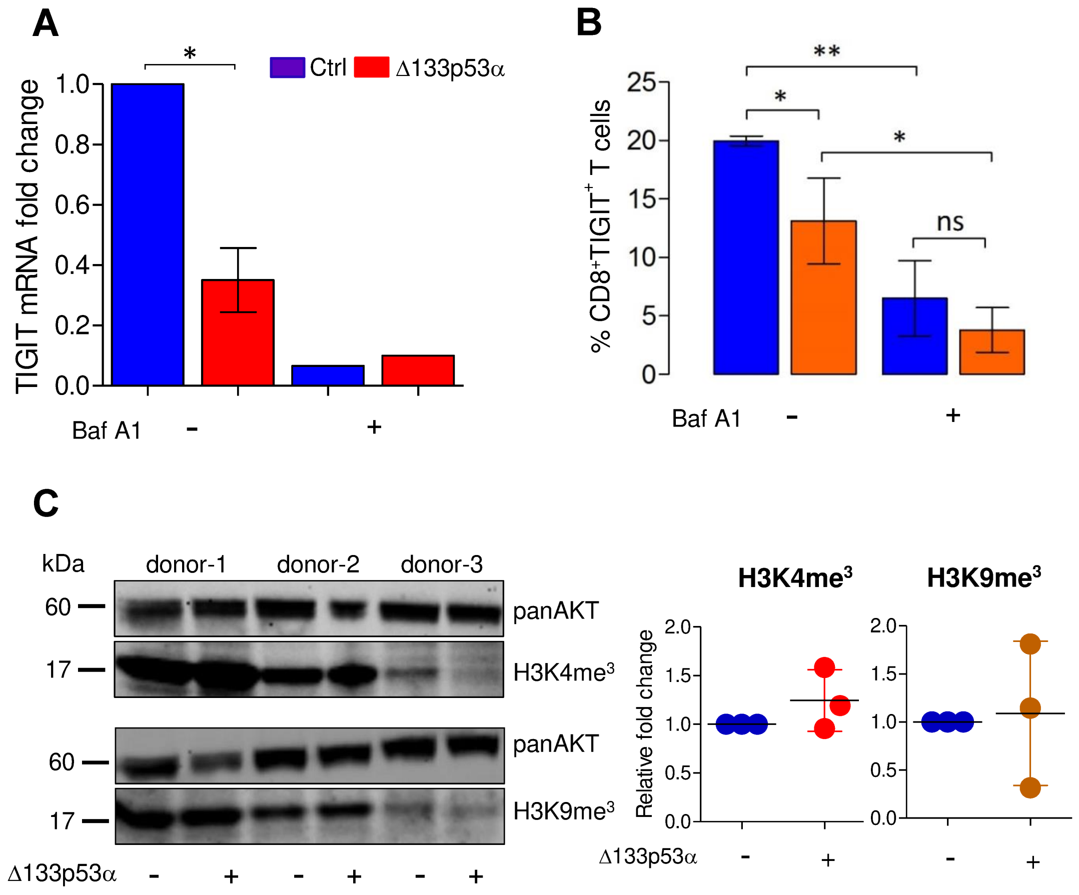

Figure S5. Δ133p53α role in the transcriptional regulation of TIGIT expression. (A) Change in fold expression of TIGIT mRNA in engineered human CD8+ T cells (1-3 weeks post transduction). T cells were treated with bafilomycin A1 for 24h and assessed by qRT-PCR for TIGIT transcripts. Error bars indicate standard error of mean (SEM) (n=3 biological replicates). One representative experiment is depicted for Baf A1 treatment. (B) Flow cytometric data showing the corresponding percentage of CD8+TIGIT+ T cells. Error bars indicate standard error of mean (SEM) (n=3 biological replicates). (C) Protein expression analysis of the histone marks H3K4me3 and H3K9me3 in engineered CD8+ T cells from three different healthy donors (western blots, left panel) and the relative fold change in expression compared to control-T cells (histogram plots, right panel). $*P < 0.05$, $**P < 0.01$, ns (not significant), by two-tailed Student's *t* test.

BMJ Publishing Group Limited (BMJ) disclaims all liability and responsibility arising from any reliance placed on this supplemental material which has been supplied by the author(s)

jitc-2020-001846.R2

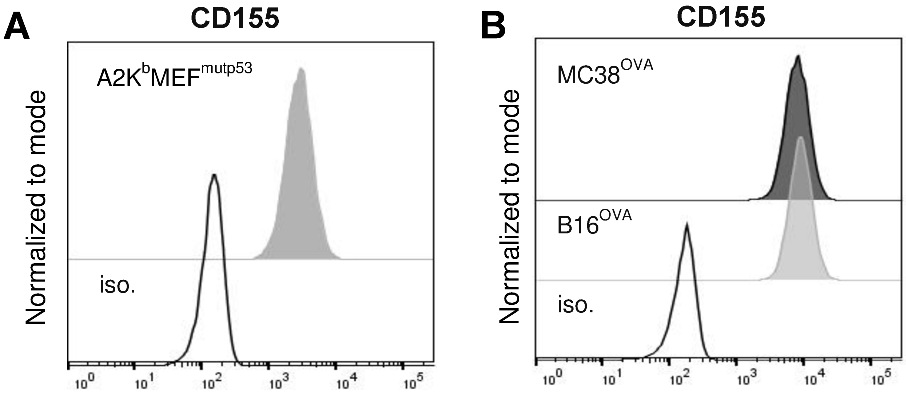

Figure S6. Expression levels of CD155 for A2K^{b}MEF^{mutp53} (A) MC38^{OVA} and B16^{OVA} (B) were analyzed by flow cytometry.

Legscha KJ, *et al. J Immunother Cancer* 2021; 9:e001846. doi: 10.1136/jitc-2020-001846

jitc-2020-001846.R2

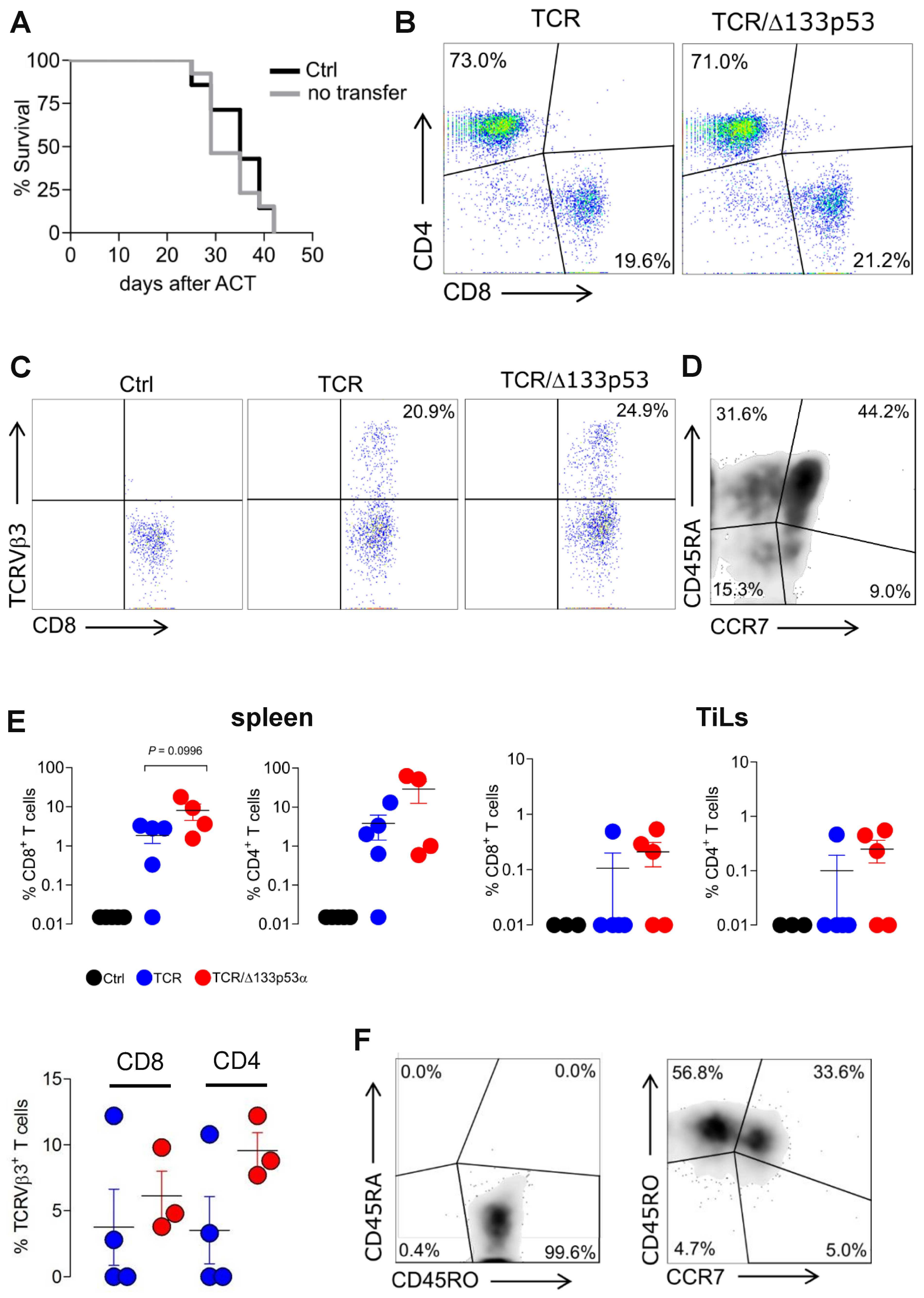

Legscha KJ, *et al. J Immunother Cancer* 2021; 9:e001846. doi: 10.1136/jitc-2020-001846

jitc-2020-001846.R2

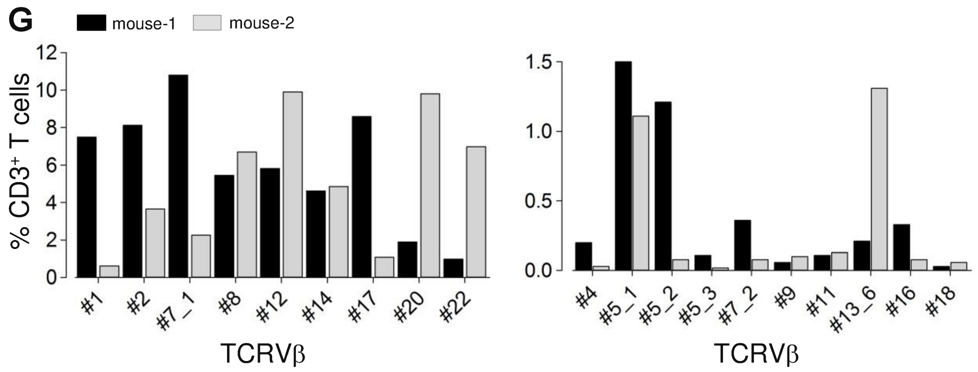

Figure S7. Phenotype and persistence of TCR/Δ133p53α-engineered T cells in vivo. (A) Survival curves for mice treated with mock-control modified T cells (Ctrl, n=13) or no transfer T cells (PBS only, n=7). Representative flow plots show comparable starting CD8/CD4 ratio (B) and TCRVβ3 expression (C) in Δ133p53α- and control-T cells at the time of infusion in mice. (D) Phenotype of engineered T cells prior adoptive transfer, determined by the expression of CD45RA and CCR7. (E) Frequency of tissue-infiltrating infused T cells as percent of CD8+ or CD4+ T cells per spleen or tumor (TiLs) in mice at day 40-60 (endpoint) after transfer. Data represent mean numbers +/- SEM from 4-5 mice in each group. The frequency of spleen-infiltrating TCR-specific CD8+ and CD4+ T cells is shown as percent of TCRVβ3^{+} T cells per tissue. (F) Phenotype of infused T cells in vivo, characterized by the expression of CD45RA, CD45RO and CCR7. (G) Analysis of the TCR Vβ repertoire of TCR-engineered T cells in vivo by flow cytometry with an antibody panel directed against 19 individual TCR/Vβ chains. Histograms depicting the frequency of high (left) and rare (right) T cell clones from isolated spleens of two representative mice.

jitc-2020-001846.R2

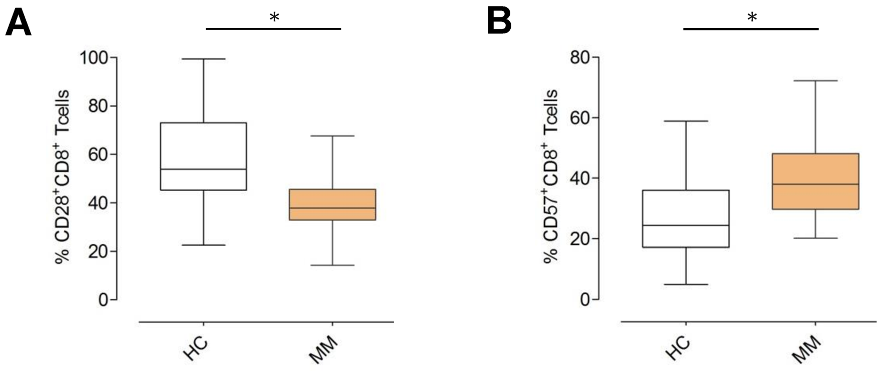

Figure S8. CD28 vs. CD57 expression profile in CD8+ T cells from multiple myeloma patients. (A) Box plots demonstrating the higher frequency of total CD28+CD8+ T cells in the peripheral blood from healthy controls (HC) compared to MM patients, as measured by flow cytometry (P = 0.0127). (B) Box plots showing the reduced frequency of total CD57+CD8+ T cells in HC compared to MM patients (P = 0.0166). HC (n = 15), MM (n = 10). P values determined by two-tailed Student's t test.

jitc-2020-001846.R2

1 **Table S1. Antibodies for flow cytometry**

antibodies	clone	source
anti-human CD3, APC	UCHT1	BD Biosciences
anti-human CD3, APC-R700	UCHT1	BD Biosciences
anti-human CD4, FITC	RPA-T4	BD Biosciences
anti-human CD45, APC-H7	2D1	BD Biosciences
anti-human CD8α, APC	RPA-T8	BD Biosciences
anti-human CD8α, FITC	HIT8a	BD Biosciences
anti-human CD27, PE	M-T271	BD Biosciences
anti-human CD27, APC	REA499	Miltenyi Biotec
anti-human CD28, FITC	CD28.2	BD Biosciences
anti-human CD28, PE	CD28.2	BD Biosciences
anti-human CD57, APC	NK-1	BD Biosciences
anti-human CD57, BV421	NK-1	BD Biosciences
anti-human CD62L, PC-5	DREG56	Immunotec
anti-human CCR7, FITC	150503	R&D Systems
anti-human PD-L1, APC	MIH1	BD Biosciences
anti-human PD-1, APC	J43	ThermoFischer Scientific
anti-human PD-1, FITC	MIH4	BD Biosciences
anti-human PD-1, BV421	EH1.21	BD Biosciences
anti-human CD160, PE	BY55	BD Biosciences
anti-human TIGIT, APC	REA1004	Miltenyi Biotec
anti-human TIGIT, PE-Cy7	A15153G	BioLegend
anti-human TIGIT, PE	MBSA43	Invitrogen
anti-human CD155, APC	300907	R&D Systems
anti-human CD155, PE	SKII.4	BD Biosciences
anti-human CD107a, PE-Cy5	H4A3	BD Biosciences
anti-human CD45RA, PE	HI100	BD Biosciences
anti-murine TCRVβ3, PE	KJ25	BD Biosciences
anti-human CD155 (neutralizing)	SKII.4	BioLegend
anti-human TIGIT (neutralizing)	MBSA43	eBioscience
anti-HLA-A2.1, PE	BB7.2	BD Biosciences
Anti-human GLUT1-Alexa Fluor® 647	202915	BD Biosciences

anti-murine TIGIT, BV421	1G9	BD Biosciences
anti-murine TIGIT, PE	REA536	Miltenyi Biotec
anti-murine PD-1, APC	J43	Invitrogen
anti-murine CD155, PE	3F1	BD Biosciences
anti-murine PD-L1, PE/Cy7	10F.9G2	BioLegend

jitc-2020-001846.R2

Table S2. Primers for qRT-PCR

**Target**	**Forward**	**Reverse**
TIGIT	TGCCAGGTTCCAGATTCCA	ACGATGACTGCTGTGCAGATG
GAPDH	GTTTACATGTTCCAATATGATTCCAC	TCATATTTGGCAGGTTTTTCTAGAC

Legscha KJ, *et al. J Immunother Cancer* 2021; 9:e001846. doi: 10.1136/jitc-2020-001846
