# Supplementary material for: Δ133p53α enhances metabolic and cellular fitness of TCR-engineered T cells and promotes superior antitumor immunity
Source: J Immunother Cancer. 2021 Jun 10;9(6):e001846. doi: 10.1136/jitc-2020-001846 (PMC8194333; doi:10.1136/jitc-2020-001846)
Supplement: Supplementary data [file jitc-2020-001846supp002.pdf]

Δ133p53α enhances metabolic and cellular fitness of TCR-engineered T cells and promotes superior antitumor immunity

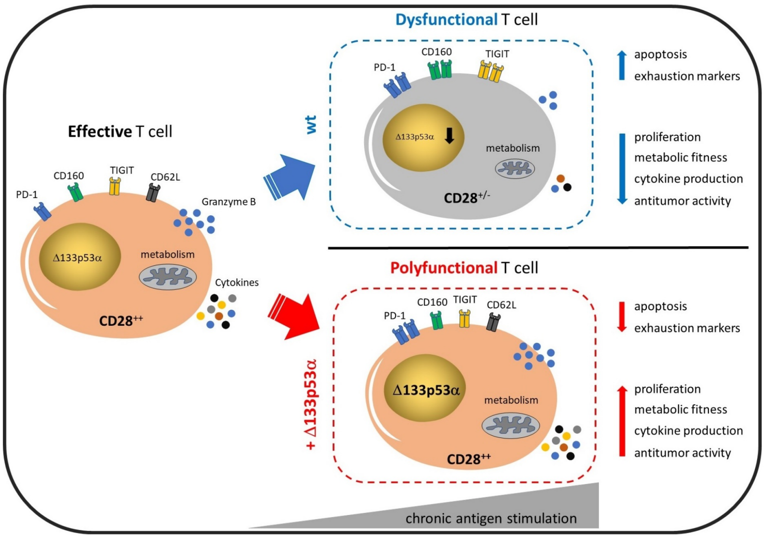

Authors

Kevin J. Legscha, Edite Antunes Ferreira, Antonios Chamoun, Alexander Lang, Mohamed HS Awwad, Gigi NHQ Ton, Danuta Galetzka, Borhane Guezguez, Michael Hundemer, Jean-Christophe Bourdon, Markus Munder, Matthias Theobald, Hakim Echchannaoui

Correspondence

echchann@uni-mainz.de

In Brief

The tumor suppressor TP53-derived isoform Δ133p53α functions as a novel modulator of metabolic and cellular fitness of TCR-engineered T cells by delaying replicative cellular senescence, and promoting superior effector functions and enhanced antitumor-specific response.
